# Supplementary material for: Validation of lipid-related therapeutic targets for coronary heart disease prevention using human genetics
Source: Nat Commun. 2021 Oct 21;12:6120. doi: 10.1038/s41467-021-25731-z (PMC8531035; doi:10.1038/s41467-021-25731-z)
Supplement: Supplementary file 1 — Supplementary Information [file 41467_2021_25731_MOESM1_ESM.pdf]

Supplementary Information

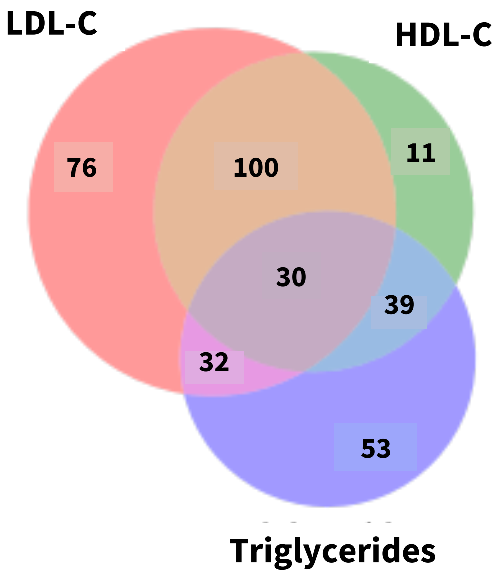

Supplementary Figure 1. **Overlap between genes encoding druggable targets associated with the major lipid subfractions.** The Venn diagram shows genes exhibiting overlapping or exclusive associations with LDL-C, HDL-C and/or TG.

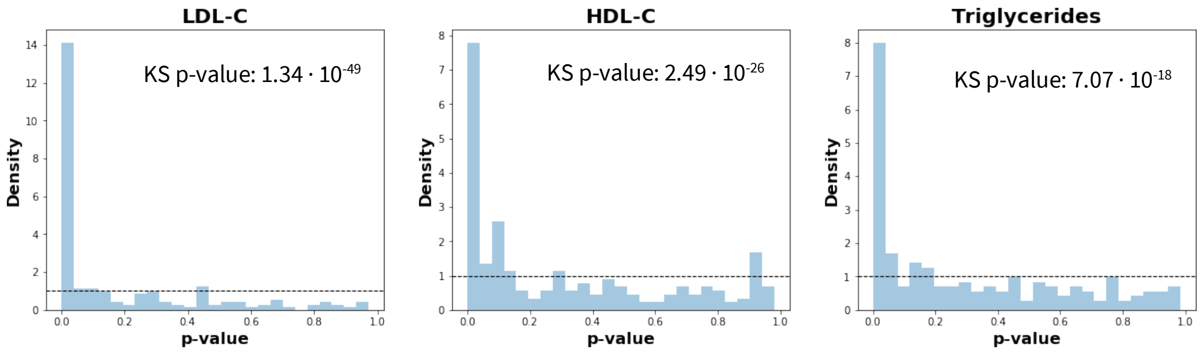

Supplementary Figure 2. Density distribution of the p-values in the discovery analysis by exposure. Kolmogorov-Smirnov (KS) goodness-of-fit test (two-sided) against the continuous uniform distribution of *P* values (black dashed line) expected under the null-hypothesis of no association between any of the targets and coronary heart disease, when the effect is instrument via LDL-C, HDL-C and TG effects.

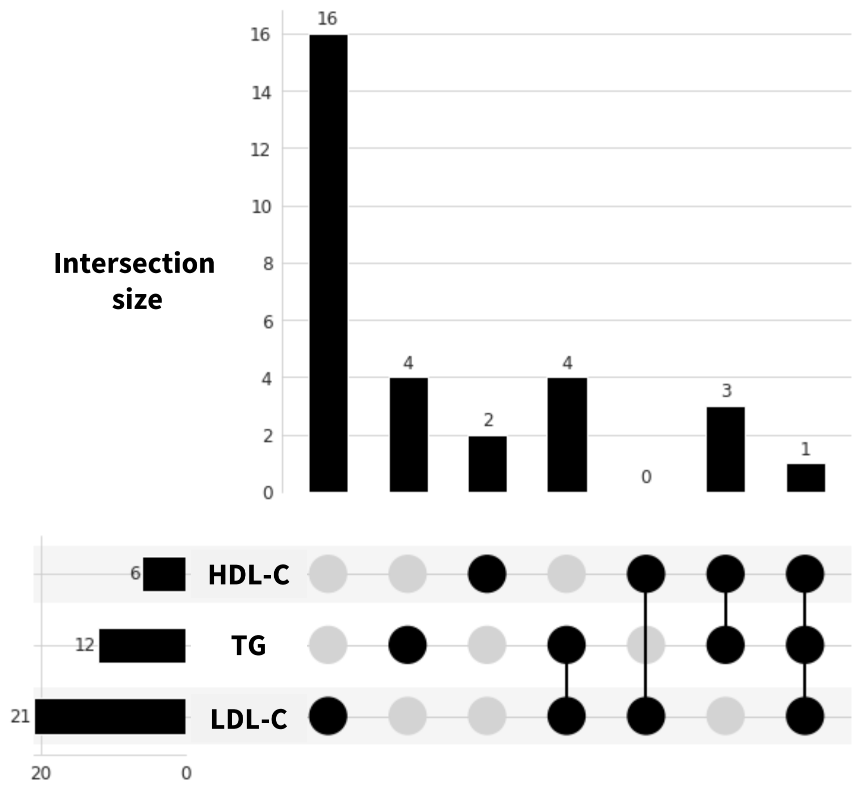

Supplementary Figure 3. The sets of assigned genes associated with LDL-C, HDL-C, TG that encode druggable targets. Genes encoding druggable targets were included if they demonstrated concordant direction of effect in the discovery and validation studies on CHD showing a causal effect of one or more lipid sub-fractions.

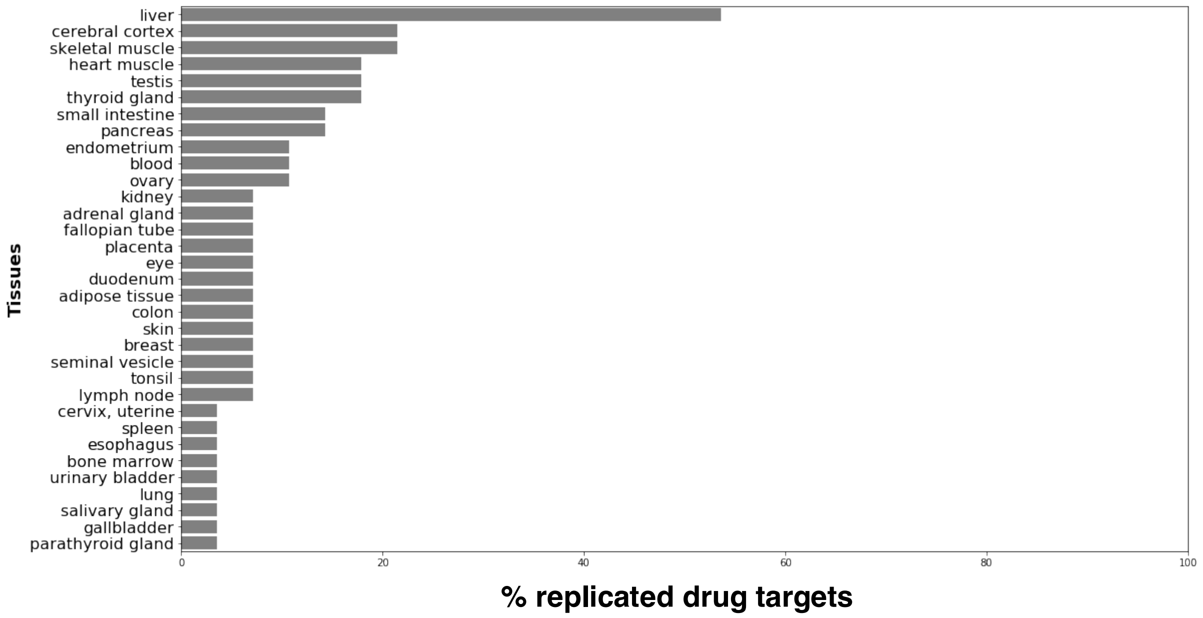

Supplementary Figure 4. Tissue expression profile of the replicated drug target genes. The tissue specificity metric, z-score, which quantifies how elevated the gene expression is in a particular tissue compared to others, was calculated for the set of 30 replicated genes encoding a druggable target. Of the 28 genes with RNAseq data available, 15 genes in the validated set (54%) were highly expressed in liver (z-score > 1), 13 (46%) did not have elevated liver expression.

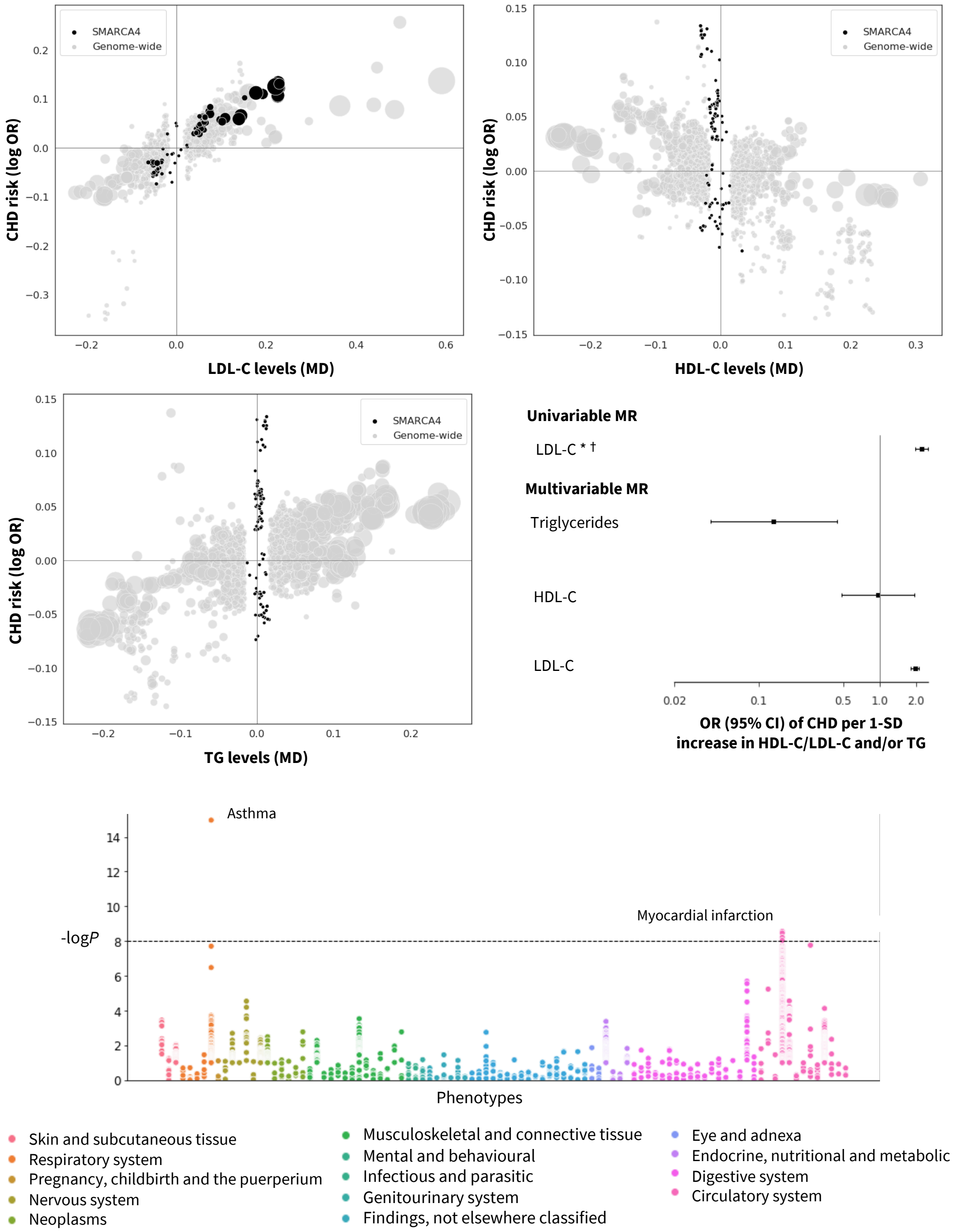

Supplementary Figure 5. Prioritized target: SMARCA4. The top and middle left panels show genetic associations at the locus (± 50kbp) in black vs genome-wide associations (grey, *P* value < $1x10^{-6}$ based on two-sided z-tests). The x-axis shows the per allele effect on the corresponding lipid expressed as mean difference (MD) from GLGC and the y-axis indicates the per allele effect on CHD expressed as log odds ratios (OR) from CardiogramPlusC4D. The marker size indicates the significance of the association with the lipid sub-fraction (*P*-value). The middle right panel shows the result of the univariable and multivariable (drug target) *cis*-MR results presented as OR and 95% confidence intervals with lipid exposure (n=188,577 individuals) and CHD outcome (n= 60,801 cases and 123,504 controls). '*' indicates the MR estimates as being replicated, and '†' that the lipid effect and CHD signals are co-localized. The bottom panel shows disease associations at the locus with 102 clinical end points from UK Biobank and GWAS Consortia.

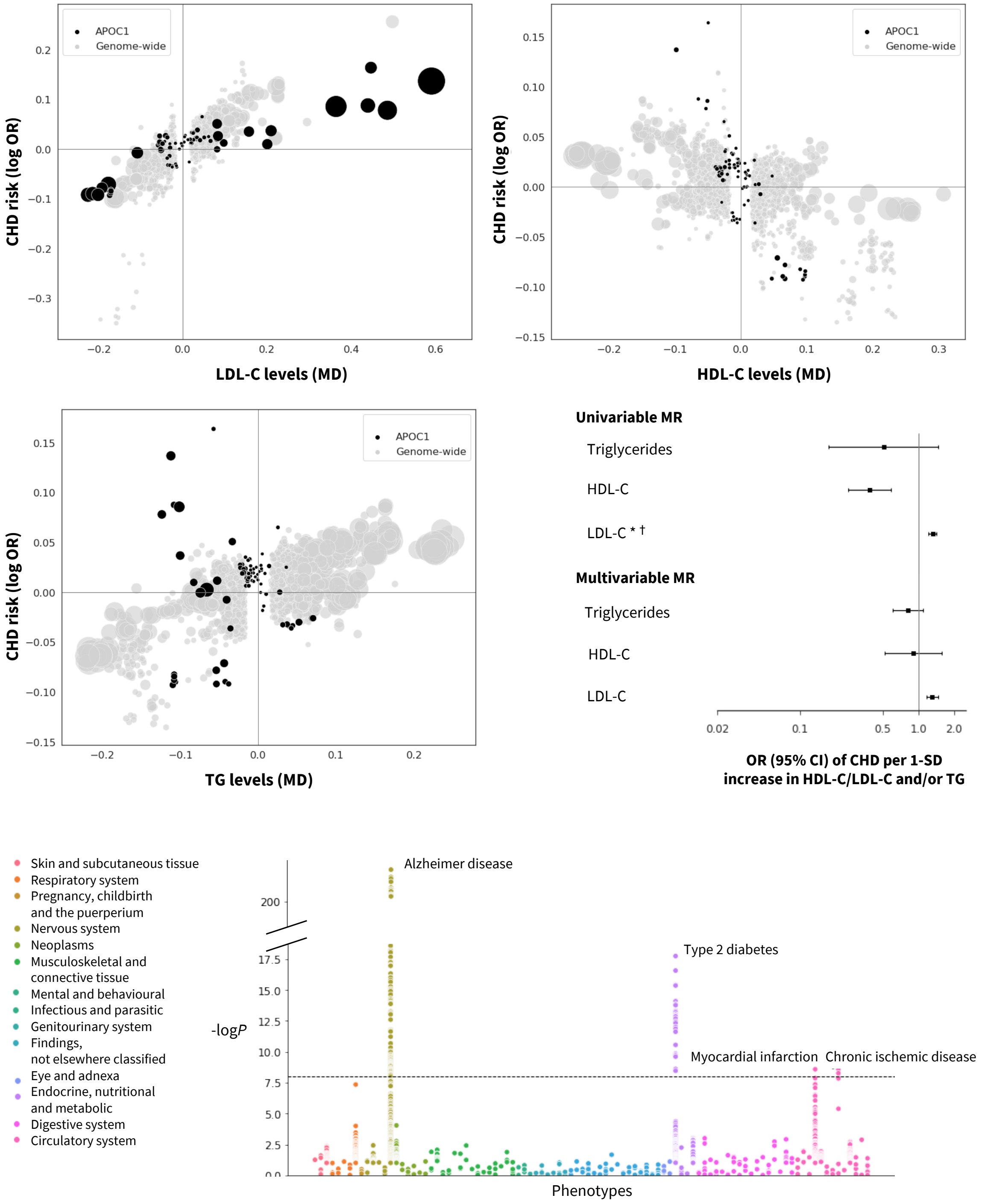

Supplementary Figure 6. Prioritized target: APOC1. The top and middle left panels show genetic associations at the locus (± 50kbp) in black vs genome-wide associations (grey, *P* value < $1x10^{-6}$ based on two-sided z-tests). The x-axis shows the per allele effect on the corresponding lipid expressed as mean difference (MD) from GLGC and the y-axis indicates the per allele effect on CHD expressed as log odds ratios (OR) from CardiogramPlusC4D. The marker size indicates the significance of the association with the lipid sub-fraction (*P*-value). The middle right panel shows the result of the univariable and multivariable (drug target) *cis*-MR results presented as OR and 95% confidence intervals with lipid exposure (n=188,577 individuals) and CHD outcome (n= 60,801 cases and 123,504 controls). '*' indicates the MR estimates as being replicated, and '†' that the lipid effect and CHD signals are co-localized. The bottom panel shows disease associations at the locus with 102 clinical end points from UK Biobank and GWAS Consortia.

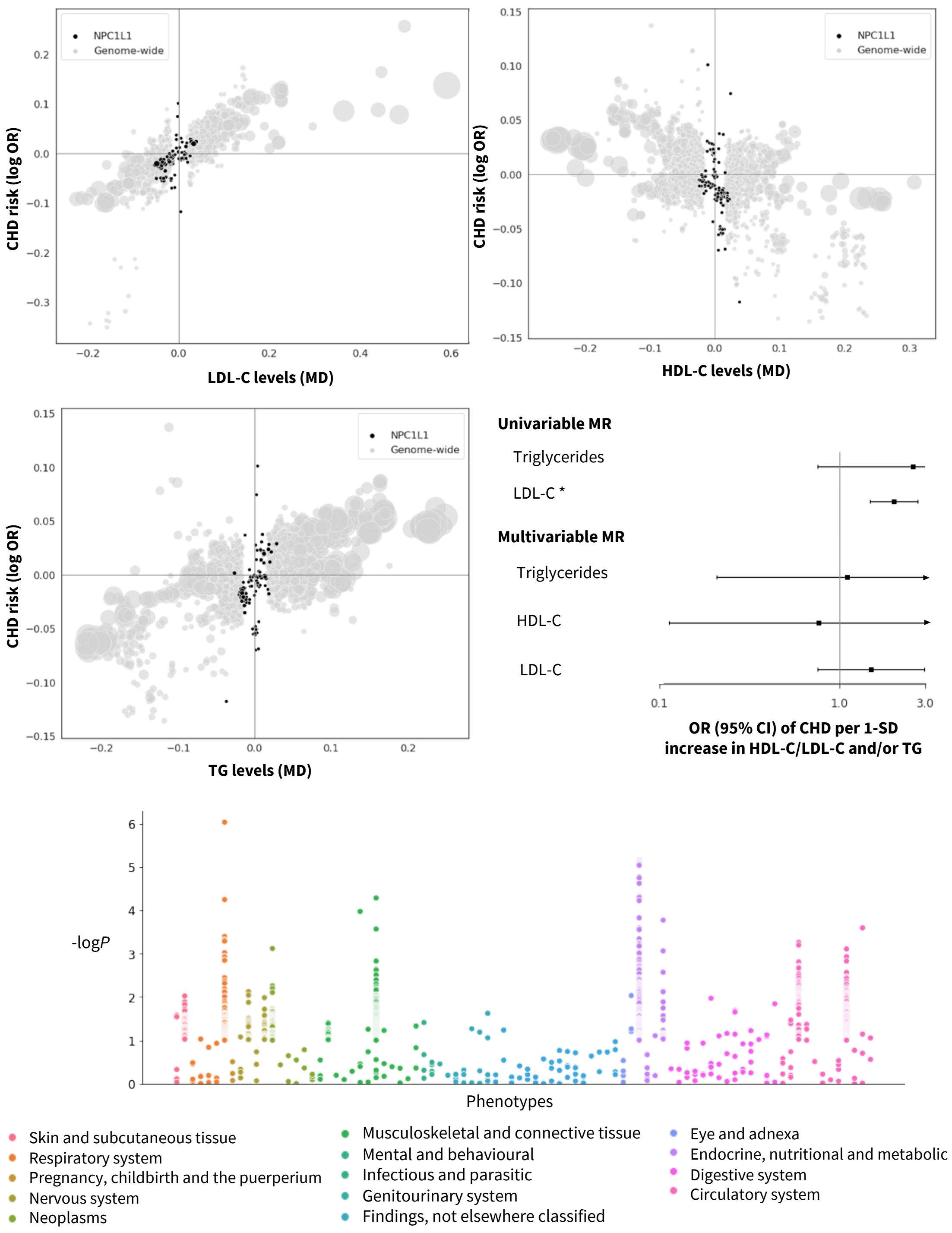

Supplementary Figure 7. Prioritized target: NPC1L1. The top and middle left panels show genetic associations at the locus (± 50kbp) in black vs genome-wide associations (grey, *P* value < 1×10^{-6} based on two-sided z-tests). The x-axis shows the per allele effect on the corresponding lipid expressed as mean difference (MD) from GLGC and the y-axis indicates the per allele effect on CHD expressed as log odds ratios (OR) from CardiogramPlusC4D. The marker size indicates the significance of the association with the lipid sub-fraction (*P*-value). The middle right panel shows the result of the univariable and multivariable (drug target) *cis*-MR results presented as OR and 95% confidence intervals with lipid exposure (n=188,577 individuals) and CHD outcome (n= 60,801 cases and 123,504 controls). '*' indicates the MR estimates as being replicated, and '†' that the lipid effect and CHD signals are co-localized. The bottom panel shows disease associations at the locus with 102 clinical end points from UK Biobank and GWAS Consortia.

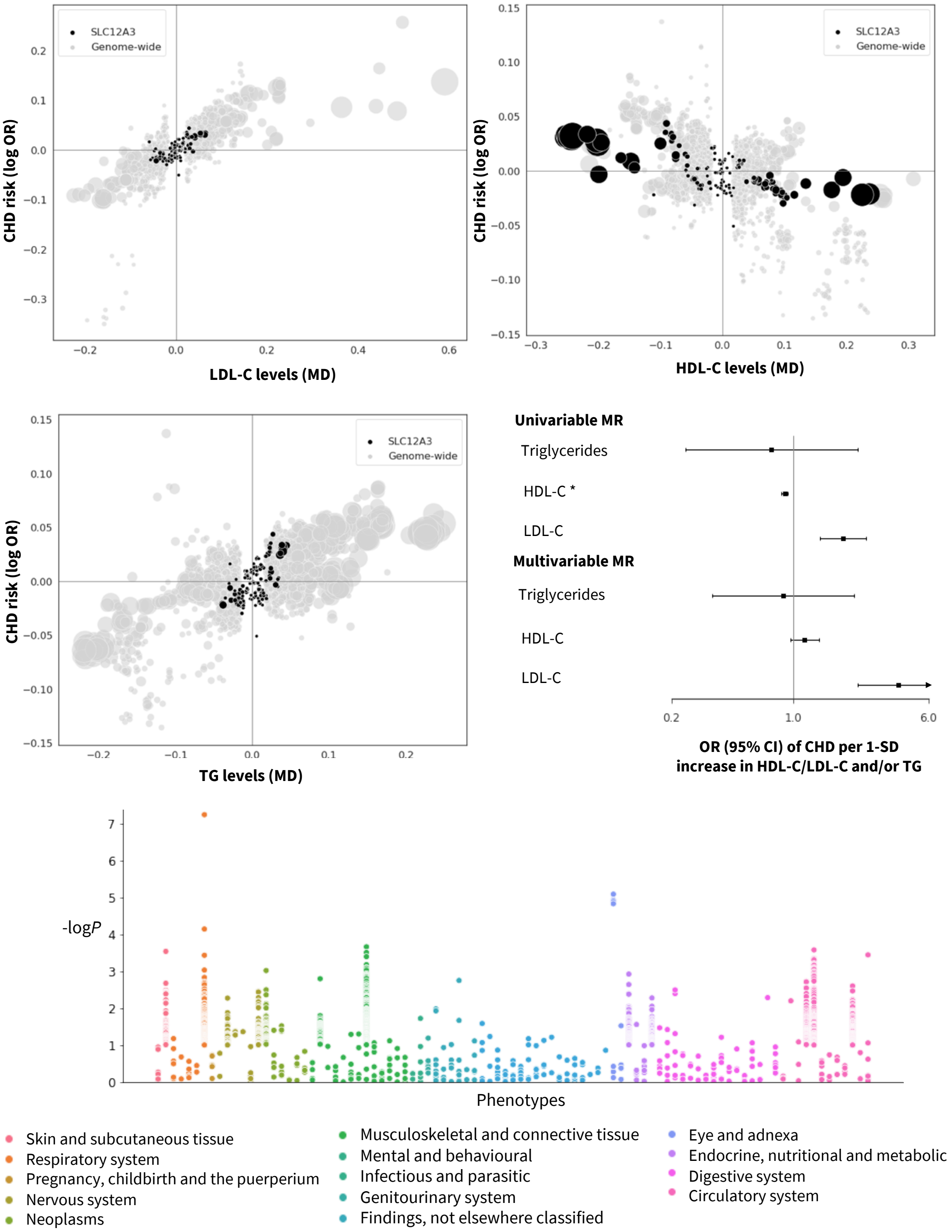

Supplementary Figure 8. Prioritized target: SLC12A3. The top and middle left panels show genetic associations at the locus (± 50kbp) in black vs genome-wide associations (grey, *P* value < 1×10^{-6} based on two-sided z-tests). The x-axis shows the per allele effect on the corresponding lipid expressed as mean difference (MD) from GLGC and the y-axis indicates the per allele effect on CHD expressed as log odds ratios (OR) from CardiogramPlusC4D. The marker size indicates the significance of the association with the lipid sub-fraction (*P*-value). The middle right panel shows the result of the univariable and multivariable (drug target) *cis*-MR results presented as OR and 95% confidence intervals with lipid exposure (n=188,577 individuals) and CHD outcome (n= 60,801 cases and 123,504 controls). '*' indicates the MR estimates as being replicated, and '†' that the lipid effect and CHD signals are co-localized. The bottom panel shows disease associations at the locus with 102 clinical end points from UK Biobank and GWAS Consortia.

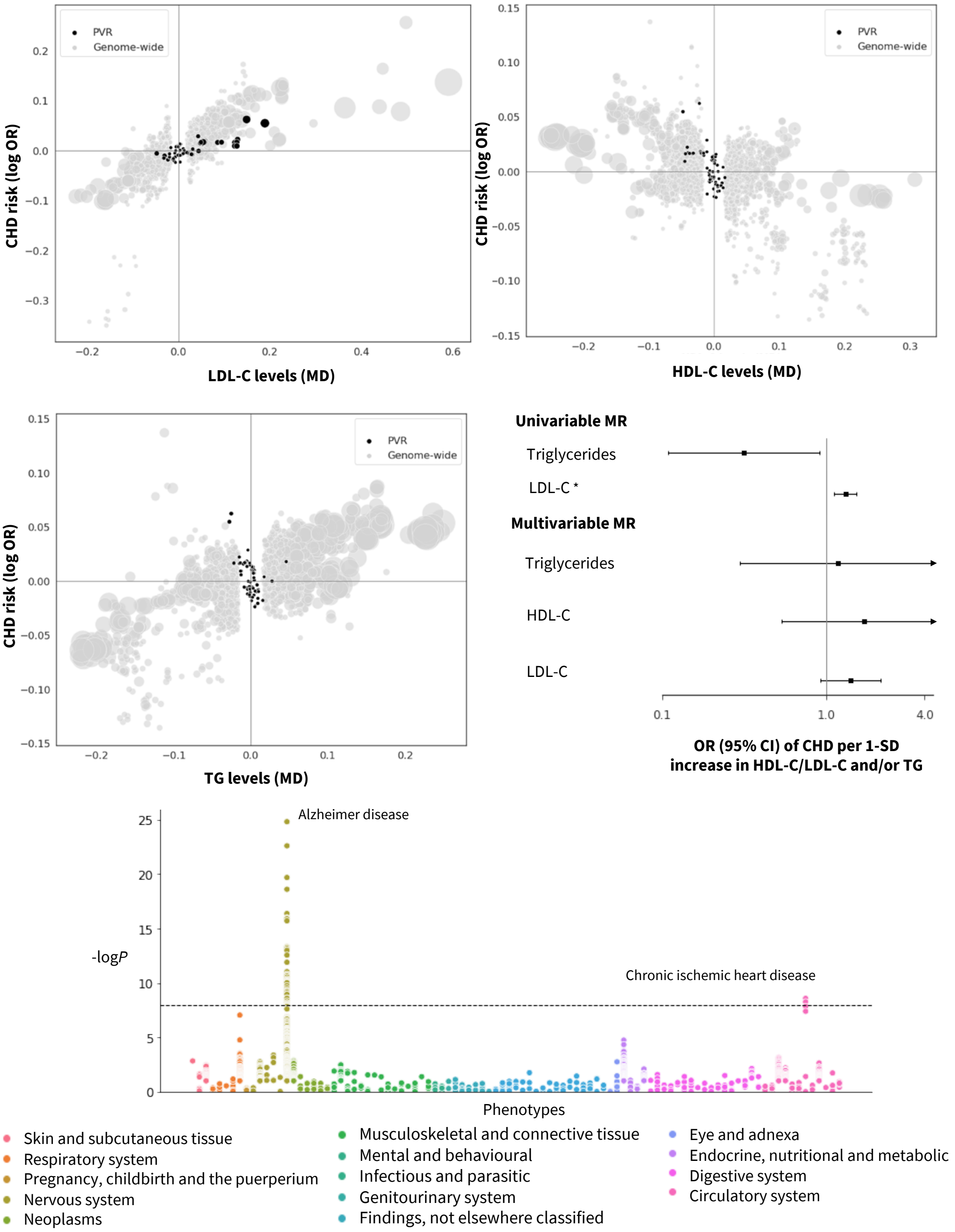

Supplementary Figure 9. Prioritized target: PVR. The top and middle left panels show genetic associations at the locus (± 50kbp) in black vs genome-wide associations (grey, *P* value < $1x10^{-6}$ based on two-sided z-tests). The x-axis shows the per allele effect on the corresponding lipid expressed as mean difference (MD) from GLGC and the y-axis indicates the per allele effect on CHD expressed as log odds ratios (OR) from CardiogramPlusC4D. The marker size indicates the significance of the association with the lipid sub-fraction (*P*-value). The middle right panel shows the result of the univariable and multivariable (drug target) *cis*-MR results presented as OR and 95% confidence intervals with lipid exposure (n=188,577 individuals) and CHD outcome (n= 60,801 cases and 123,504 controls). '*' indicates the MR estimates as being replicated, and '†' that the lipid effect and CHD signals are co-localized. The bottom panel shows disease associations at the locus with 102 clinical end points from UK Biobank and GWAS Consortia.

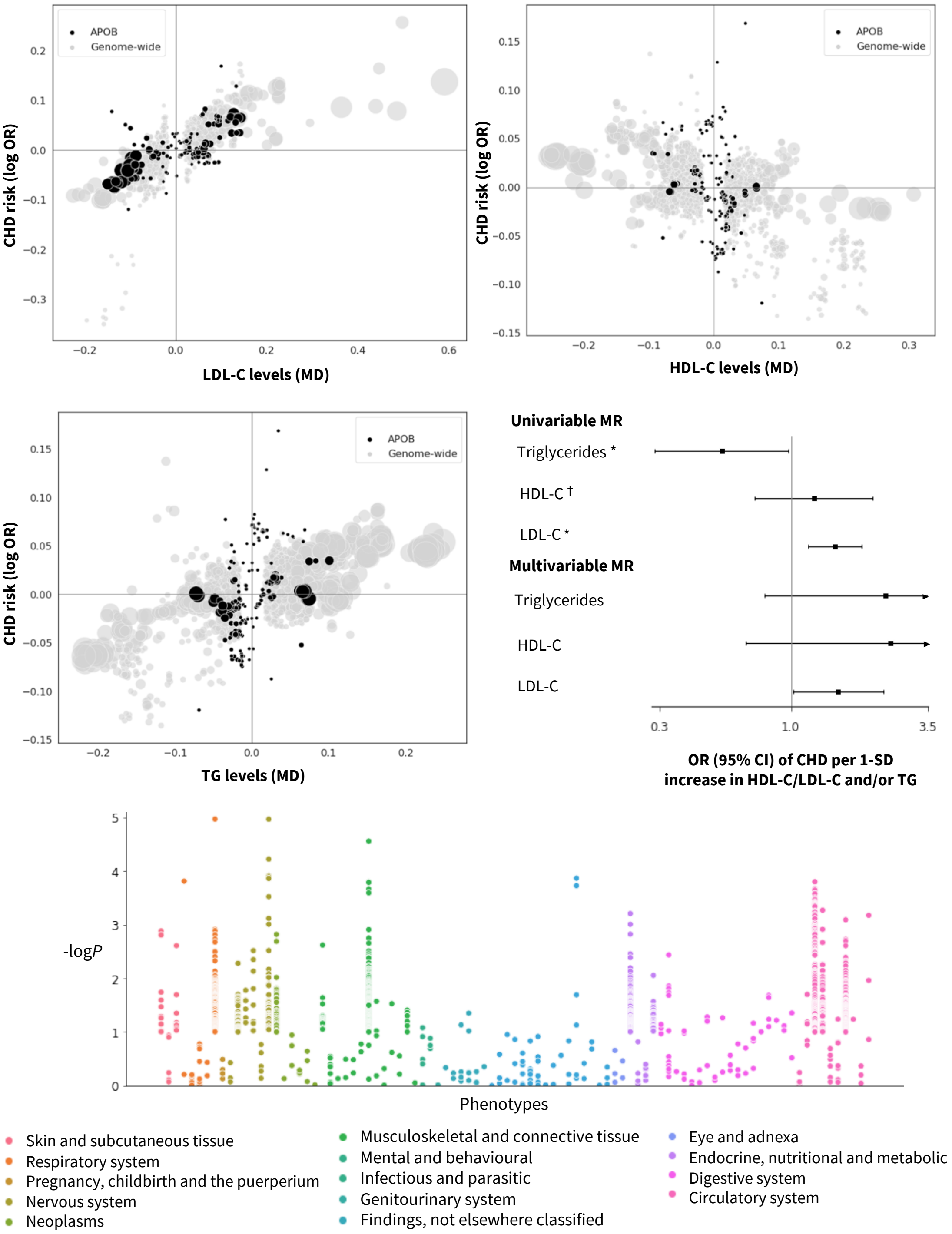

Supplementary Figure 10. Prioritized target: APOB. The top and middle left panels show genetic associations at the locus (± 50kbp) in black vs genome-wide associations (grey, *P* value < 1x10^{-6} based on two-sided z-tests). The x-axis shows the per allele effect on the corresponding lipid expressed as mean difference (MD) from GLGC and the y-axis indicates the per allele effect on CHD expressed as log odds ratios (OR) from CardiogramPlusC4D. The marker size indicates the significance of the association with the lipid sub-fraction (*P*-value). The middle right panel shows the result of the univariable and multivariable (drug target) *cis*-MR results presented as OR and 95% confidence intervals with lipid exposure (n=188,577 individuals) and CHD outcome (n= 60,801 cases and 123,504 controls). '*' indicates the MR estimates as being replicated, and '†' that the lipid effect and CHD signals are co-localized. The bottom panel shows disease associations at the locus with 102 clinical end points from UK Biobank and GWAS Consortia.

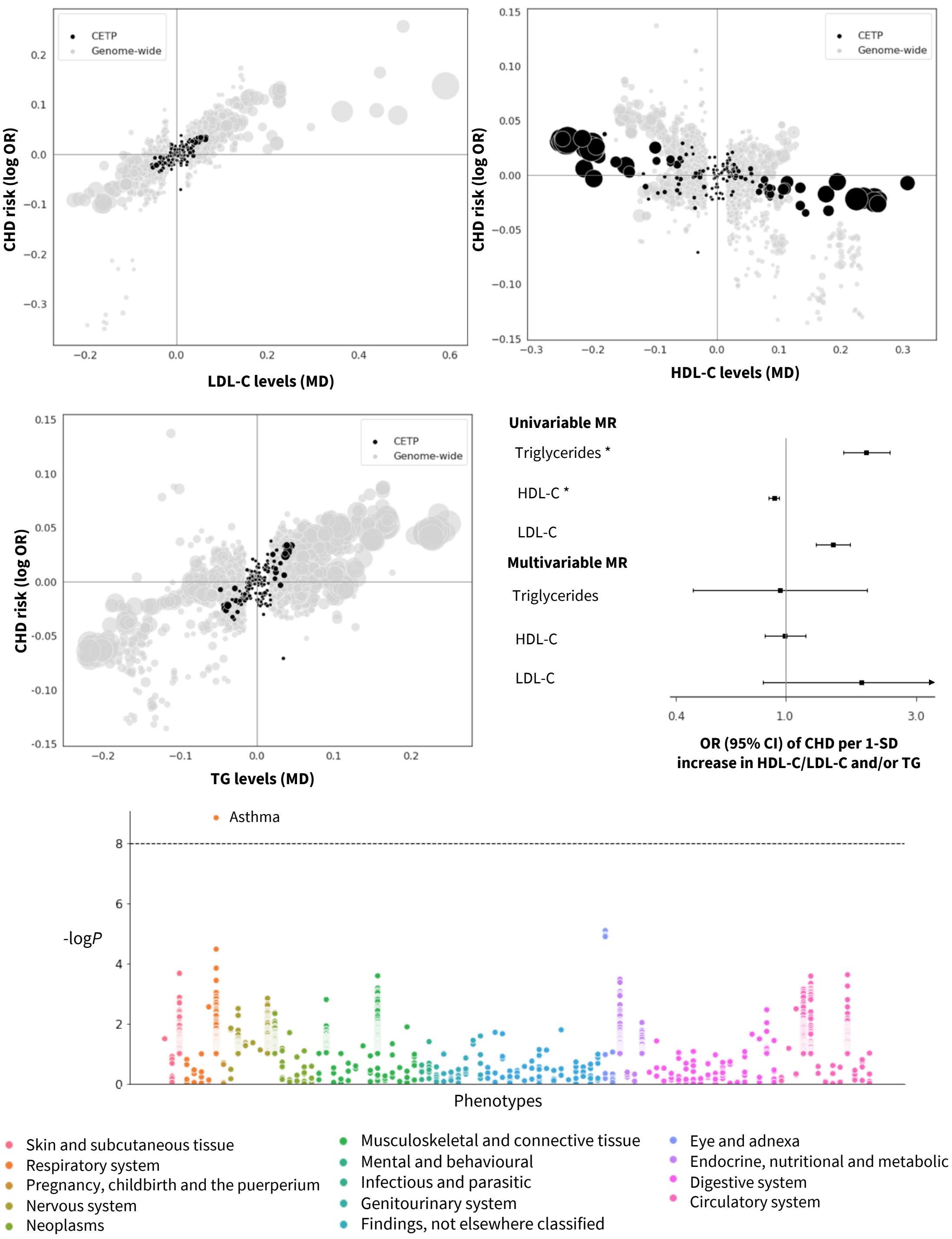

Supplementary Figure 11. Prioritized target: CETP. The top and middle left panels show genetic associations at the locus (± 50kbp) in black vs genome-wide associations (grey, *P* value < $1x10^{-6}$ based on two-sided z-tests). The x-axis shows the per allele effect on the corresponding lipid expressed as mean difference (MD) from GLGC and the y-axis indicates the per allele effect on CHD expressed as log odds ratios (OR) from CardiogramPlusC4D. The marker size indicates the significance of the association with the lipid sub-fraction (*P*-value). The middle right panel shows the result of the univariable and multivariable (drug target) *cis*-MR results presented as OR and 95% confidence intervals with lipid exposure (n=188,577 individuals) and CHD outcome (n= 60,801 cases and 123,504 controls). '*' indicates the MR estimates as being replicated, and '†' that the lipid effect and CHD signals are co-localized. The bottom panel shows disease associations at the locus with 102 clinical end points from UK Biobank and GWAS Consortia.

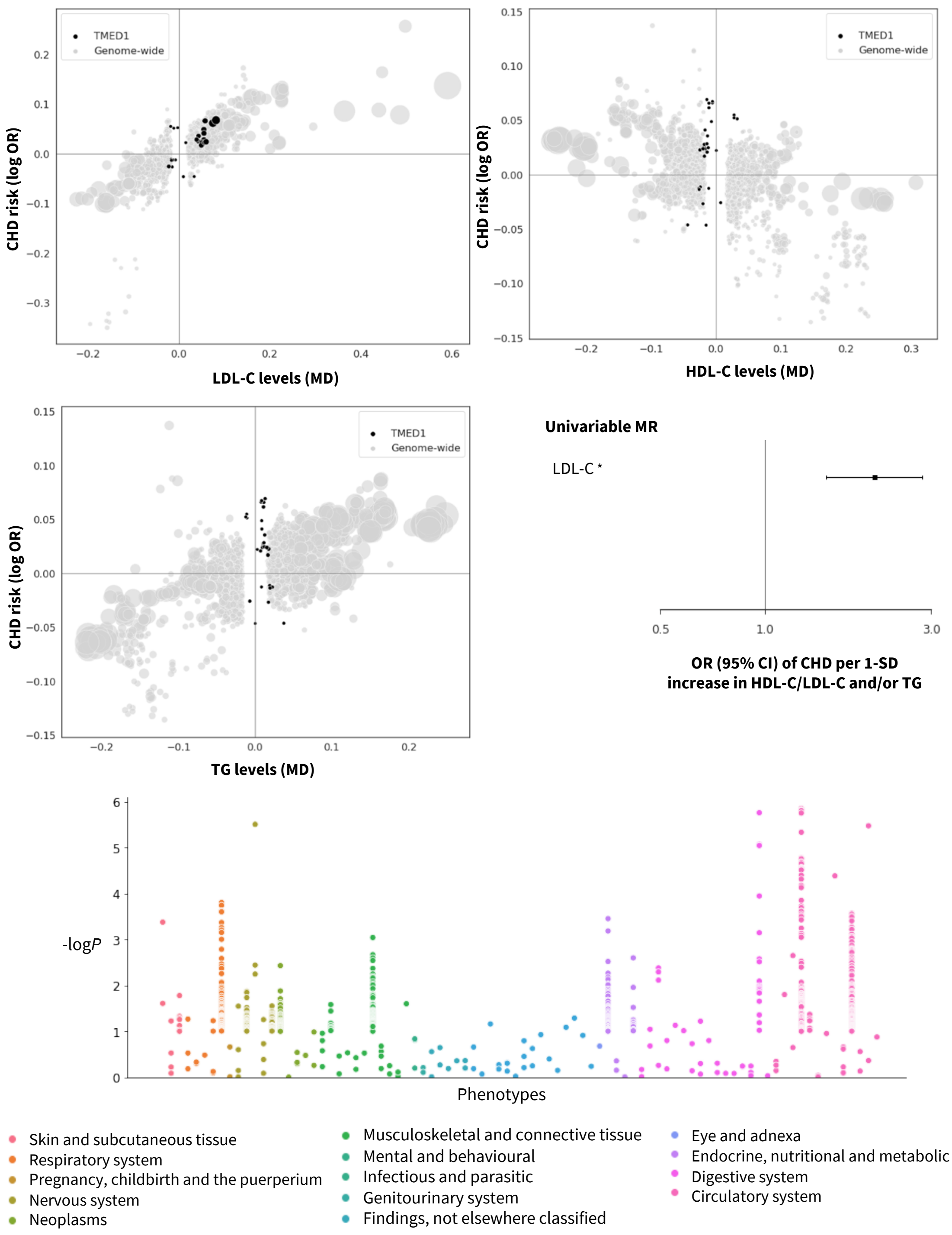

Supplementary Figure 12. Prioritized target: TMED1. The top and middle left panels show genetic associations at the locus (± 50kbp) in black vs genome-wide associations (grey, *P* value < $1x10^{-6}$ based on two-sided z-tests). The x-axis shows the per allele effect on the corresponding lipid expressed as mean difference (MD) from GLGC and the y-axis indicates the per allele effect on CHD expressed as log odds ratios (OR) from CardiogramPlusC4D. The marker size indicates the significance of the association with the lipid sub-fraction (*P*-value). The middle right panel shows the result of the univariable and multivariable (drug target) *cis*-MR results presented as OR and 95% confidence intervals with lipid exposure (n=188,577 individuals) and CHD outcome (n= 60,801 cases and 123,504 controls). '*' indicates the MR estimates as being replicated, and '†' that the lipid effect and CHD signals are co-localized. The bottom panel shows disease associations at the locus with 102 clinical end points from UK Biobank and GWAS Consortia.

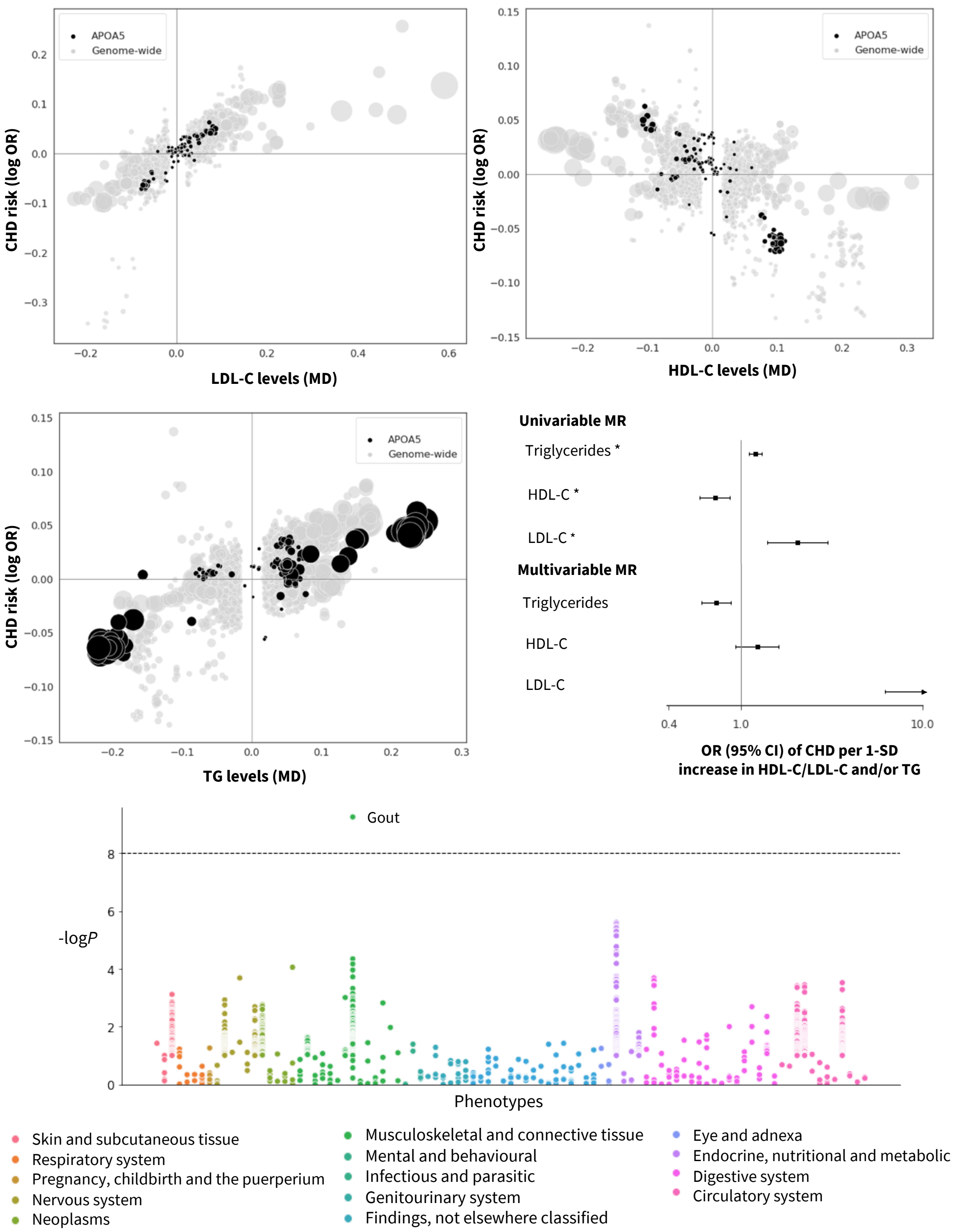

Supplementary Figure 13. Prioritized target: APOA5. The top and middle left panels show genetic associations at the locus (± 50kbp) in black vs genome-wide associations (grey, *P* value < $1x10^{-6}$ based on two-sided z-tests). The x-axis shows the per allele effect on the corresponding lipid expressed as mean difference (MD) from GLGC and the y-axis indicates the per allele effect on CHD expressed as log odds ratios (OR) from CardiogramPlusC4D. The marker size indicates the significance of the association with the lipid sub-fraction (*P*-value). The middle right panel shows the result of the univariable and multivariable (drug target) *cis*-MR results presented as OR and 95% confidence intervals with lipid exposure (n=188,577 individuals) and CHD outcome (n= 60,801 cases and 123,504 controls). '*' indicates the MR estimates as being replicated, and '†' that the lipid effect and CHD signals are co-localized. The bottom panel shows disease associations at the locus with 102 clinical end points from UK Biobank and GWAS Consortia.

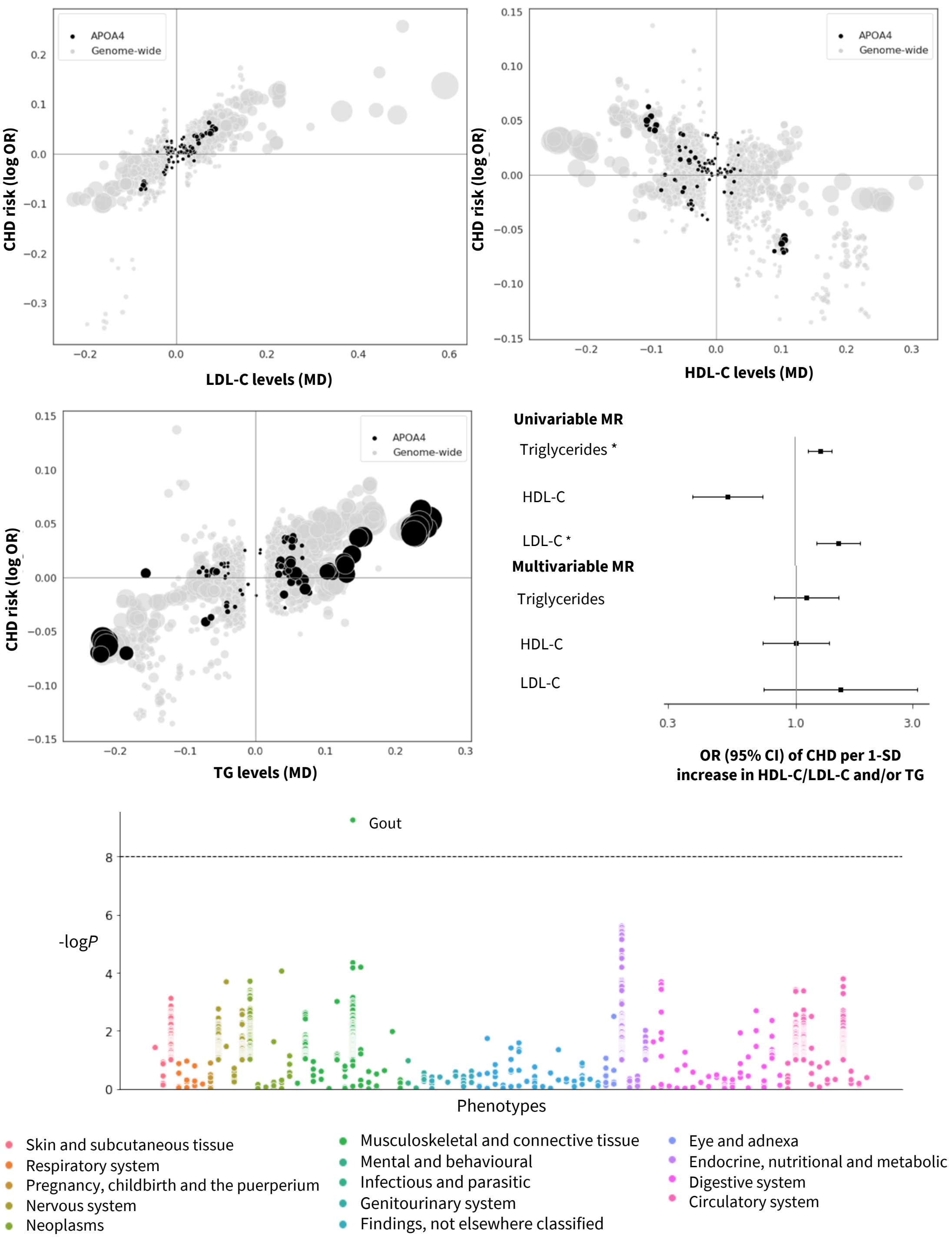

Supplementary Figure 14. Prioritized target: APOA4. The top and middle left panels show genetic associations at the locus (± 50kbp) in black vs genome-wide associations (grey, *P* value < 1×10^{-6} based on two-sided z-tests). The x-axis shows the per allele effect on the corresponding lipid expressed as mean difference (MD) from GLGC and the y-axis indicates the per allele effect on CHD expressed as log odds ratios (OR) from CardiogramPlusC4D. The marker size indicates the significance of the association with the lipid sub-fraction (*P*-value). The middle right panel shows the result of the univariable and multivariable (drug target) *cis*-MR results presented as OR and 95% confidence intervals with lipid exposure (n=188,577 individuals) and CHD outcome (n= 60,801 cases and 123,504 controls). '*' indicates the MR estimates as being replicated, and '†' that the lipid effect and CHD signals are co-localized. The bottom panel shows disease associations at the locus with 102 clinical end points from UK Biobank and GWAS Consortia.

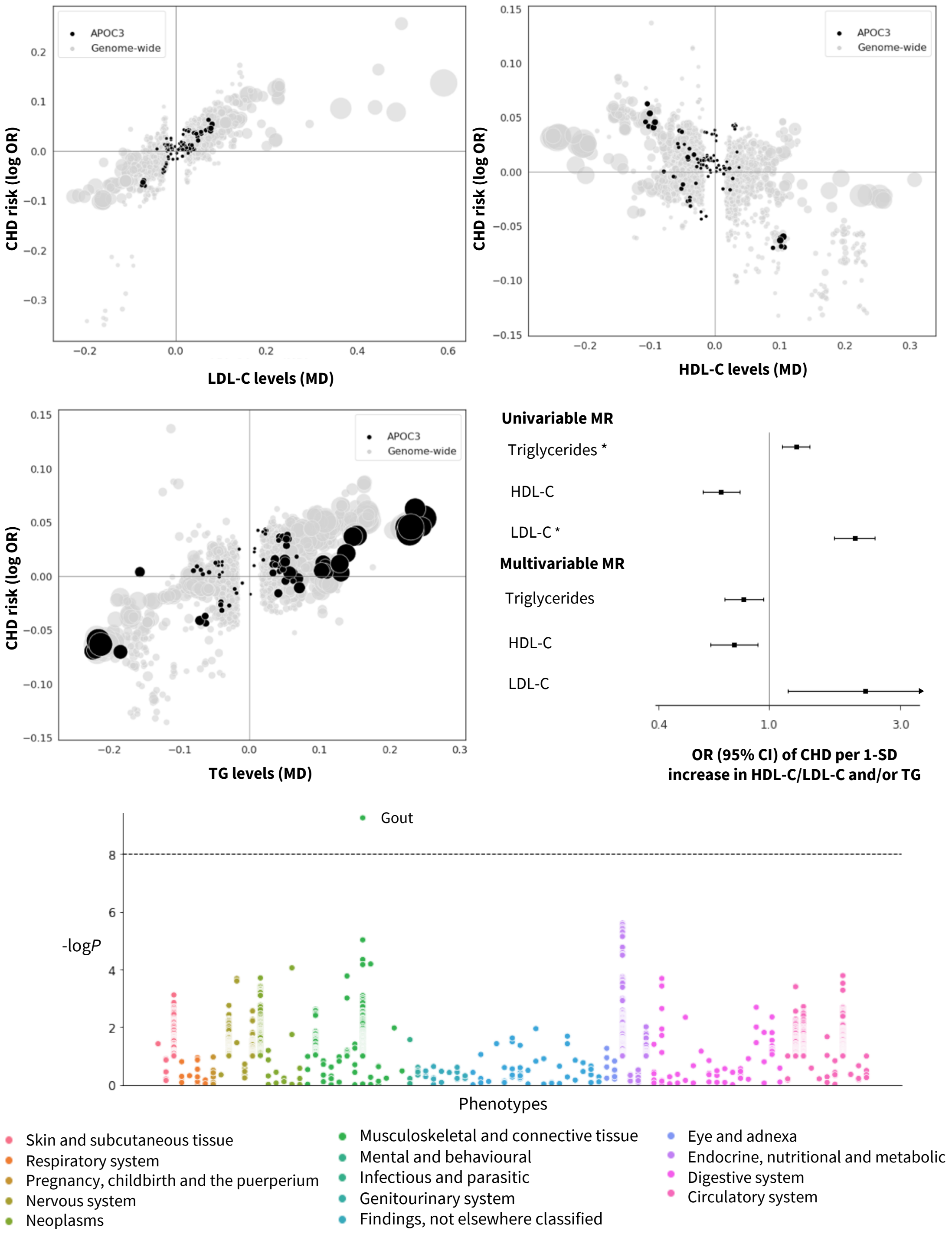

Supplementary Figure 15. Prioritized target: APOC3. The top and middle left panels show genetic associations at the locus (± 50kbp) in black vs genome-wide associations (grey, *P* value < $1x10^{-6}$ based on two-sided z-tests). The x-axis shows the per allele effect on the corresponding lipid expressed as mean difference (MD) from GLGC and the y-axis indicates the per allele effect on CHD expressed as log odds ratios (OR) from CardiogramPlusC4D. The marker size indicates the significance of the association with the lipid sub-fraction (*P*-value). The middle right panel shows the result of the univariable and multivariable (drug target) *cis*-MR results presented as OR and 95% confidence intervals with lipid exposure (n=188,577 individuals) and CHD outcome (n= 60,801 cases and 123,504 controls). '*' indicates the MR estimates as being replicated, and '†' that the lipid effect and CHD signals are co-localized. The bottom panel shows disease associations at the locus with 102 clinical end points from UK Biobank and GWAS Consortia.

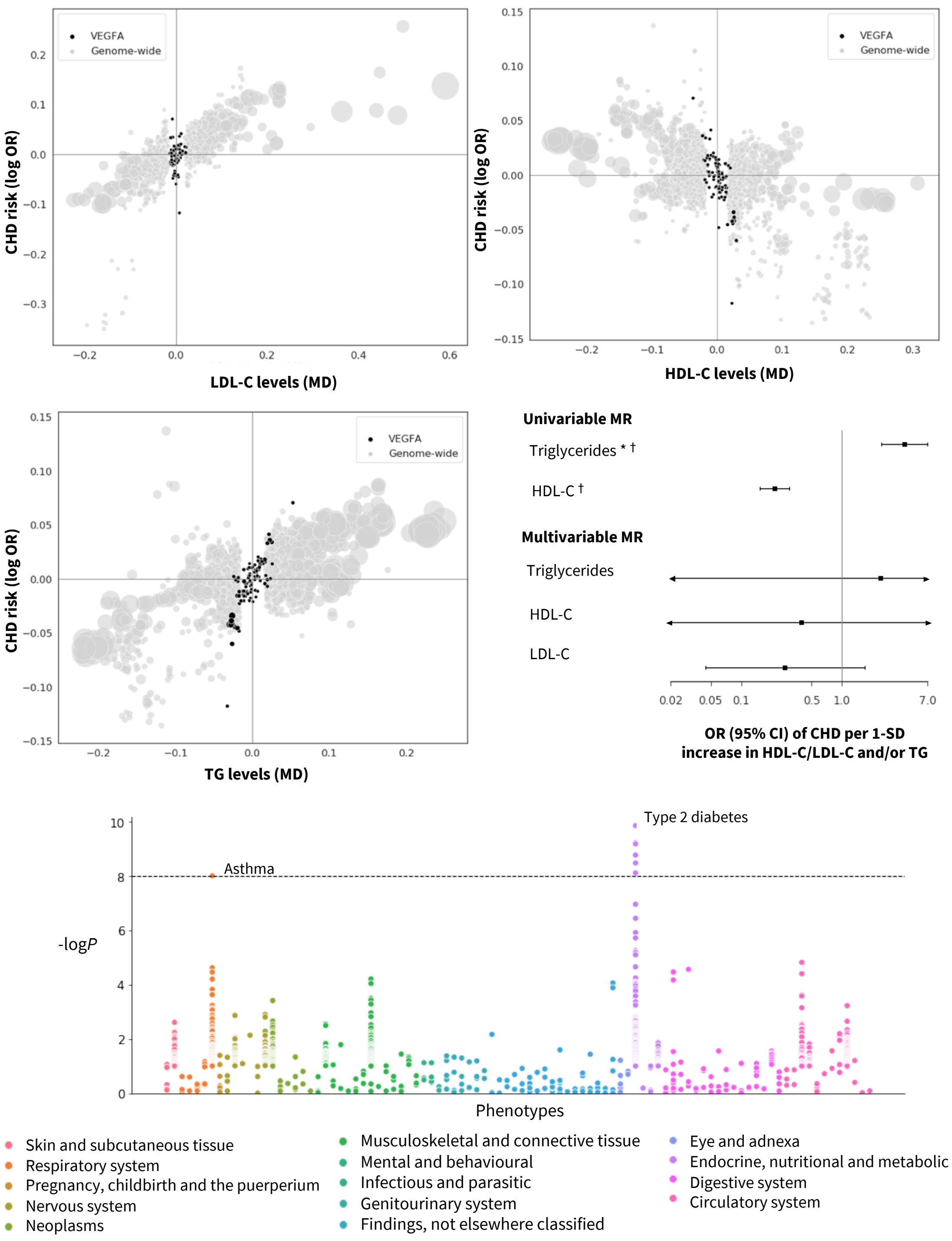

Supplementary Figure 16. Prioritized target: VEGFA. The top and middle left panels show genetic associations at the locus (± 50kbp) in black vs genome-wide associations (grey, *P* value < 1×10^{-6} based on two-sided z-tests). The x-axis shows the per allele effect on the corresponding lipid expressed as mean difference (MD) from GLGC and the y-axis indicates the per allele effect on CHD expressed as log odds ratios (OR) from CardiogramPlusC4D. The marker size indicates the significance of the association with the lipid sub-fraction (*P*-value). The middle right panel shows the result of the univariable and multivariable (drug target) *cis*-MR results presented as OR and 95% confidence intervals with lipid exposure (n=188,577 individuals) and CHD outcome (n= 60,801 cases and 123,504 controls). '*' indicates the MR estimates as being replicated, and '†' that the lipid effect and CHD signals are co-localized. The bottom panel shows disease associations at the locus with 102 clinical end points from UK Biobank and GWAS Consortia.

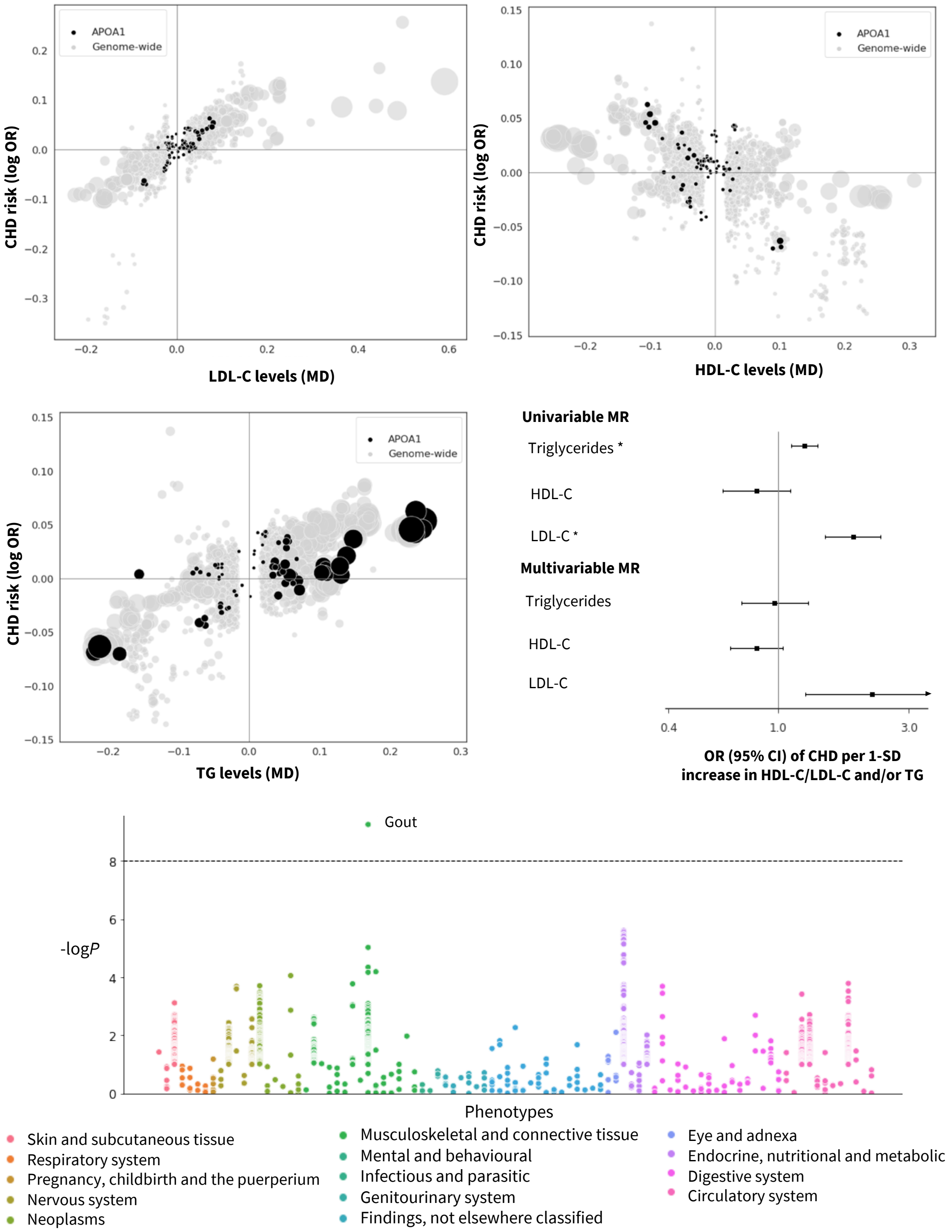

Supplementary Figure 17. Prioritized target: APOA1. The top and middle left panels show genetic associations at the locus (± 50kbp) in black vs genome-wide associations (grey, *P* value < 1×10^{-6} based on two-sided z-tests). The x-axis shows the per allele effect on the corresponding lipid expressed as mean difference (MD) from GLGC and the y-axis indicates the per allele effect on CHD expressed as log odds ratios (OR) from CardiogramPlusC4D. The marker size indicates the significance of the association with the lipid sub-fraction (*P*-value). The middle right panel shows the result of the univariable and multivariable (drug target) *cis*-MR results presented as OR and 95% confidence intervals with lipid exposure (n=188,577 individuals) and CHD outcome (n= 60,801 cases and 123,504 controls). '*' indicates the MR estimates as being replicated, and '†' that the lipid effect and CHD signals are co-localized. The bottom panel shows disease associations at the locus with 102 clinical end points from UK Biobank and GWAS Consortia.

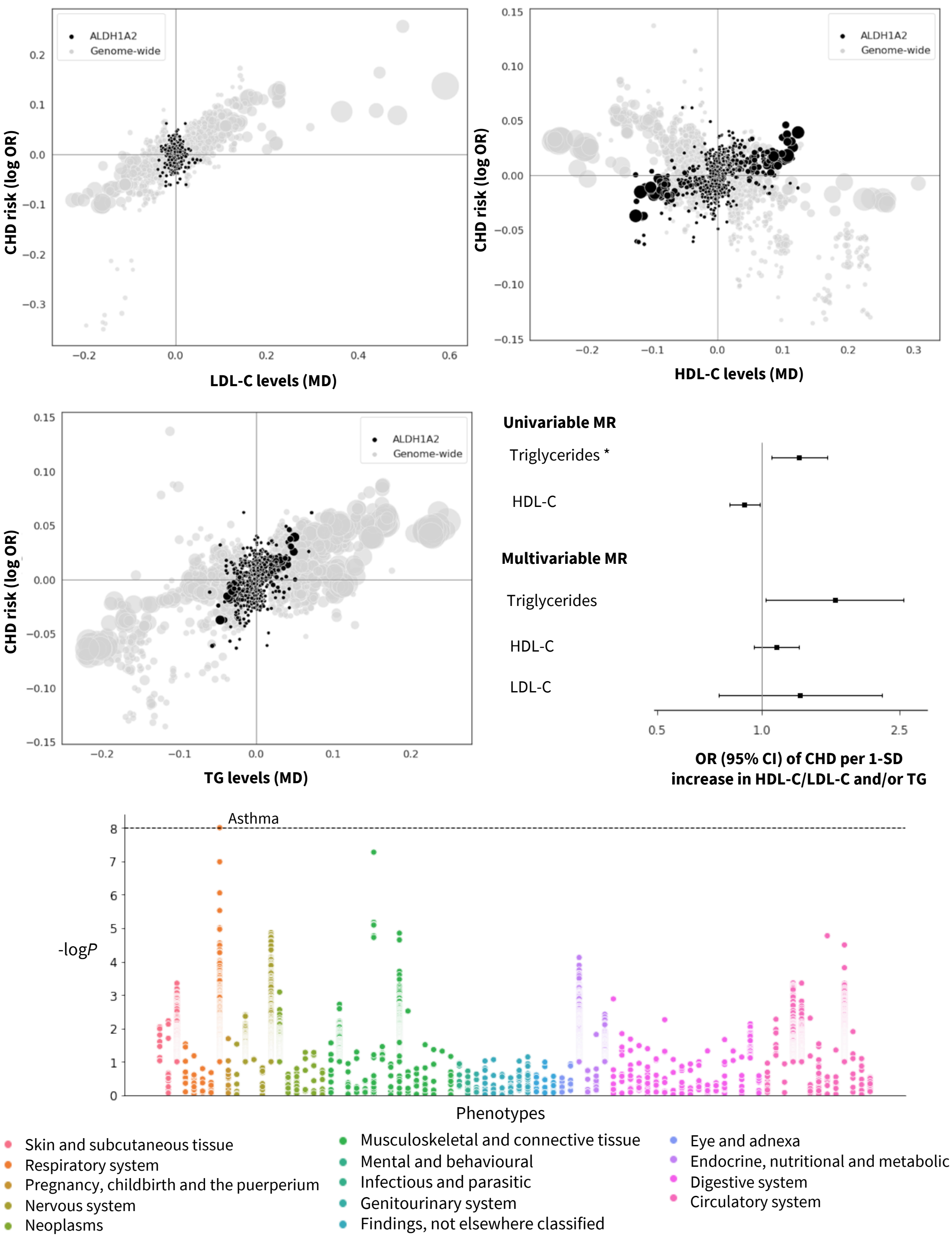

Supplementary Figure 18. Prioritized target: ALDH1A2. The top and middle left panels show genetic associations at the locus (± 50kbp) in black vs genome-wide associations (grey, *P* value < $1x10^{-6}$ based on two-sided z-tests). The x-axis shows the per allele effect on the corresponding lipid expressed as mean difference (MD) from GLGC and the y-axis indicates the per allele effect on CHD expressed as log odds ratios (OR) from CardiogramPlusC4D. The marker size indicates the significance of the association with the lipid sub-fraction (*P*-value). The middle right panel shows the result of the univariable and multivariable (drug target) *cis*-MR results presented as OR and 95% confidence intervals with lipid exposure (n=188,577 individuals) and CHD outcome (n= 60,801 cases and 123,504 controls). '*' indicates the MR estimates as being replicated, and '†' that the lipid effect and CHD signals are co-localized. The bottom panel shows disease associations at the locus with 102 clinical end points from UK Biobank and GWAS Consortia.

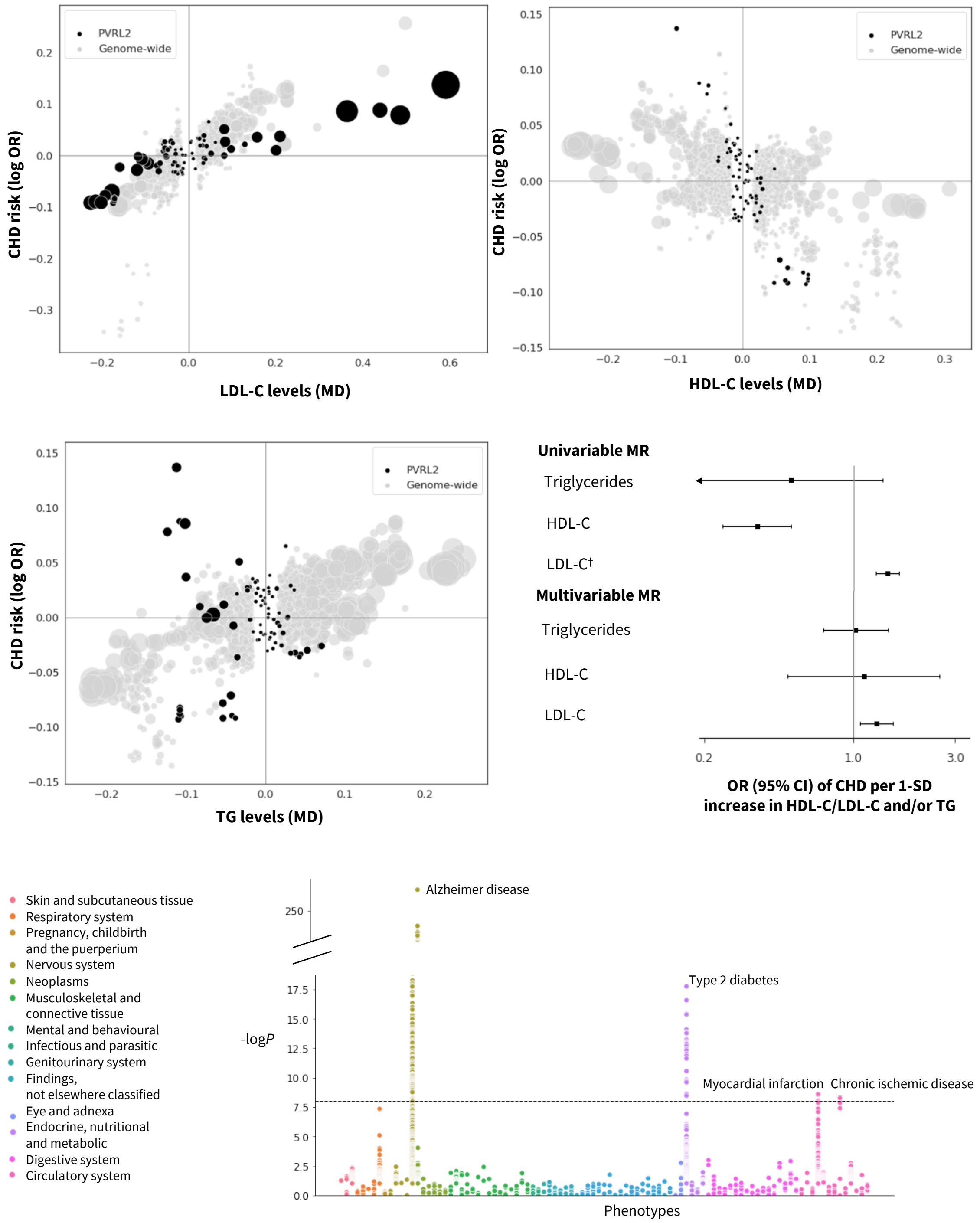

Supplementary Figure 19. Prioritized target: PVRL2. The top and middle left panels show genetic associations at the locus (± 50kbp) in black vs genome-wide associations (grey, *P* value < $1x10^{-6}$ based on two-sided z-tests). The x-axis shows the per allele effect on the corresponding lipid expressed as mean difference (MD) from GLGC and the y-axis indicates the per allele effect on CHD expressed as log odds ratios (OR) from CardiogramPlusC4D. The marker size indicates the significance of the association with the lipid sub-fraction (*P*-value). The middle right panel shows the result of the univariable and multivariable (drug target) *cis*-MR results presented as OR and 95% confidence intervals with lipid exposure (n=188,577 individuals) and CHD outcome (n= 60,801 cases and 123,504 controls). '*' indicates the MR estimates as being replicated, and '†' that the lipid effect and CHD signals are co-localized. The bottom panel shows disease associations at the locus with 102 clinical end points from UK Biobank and GWAS Consortia.

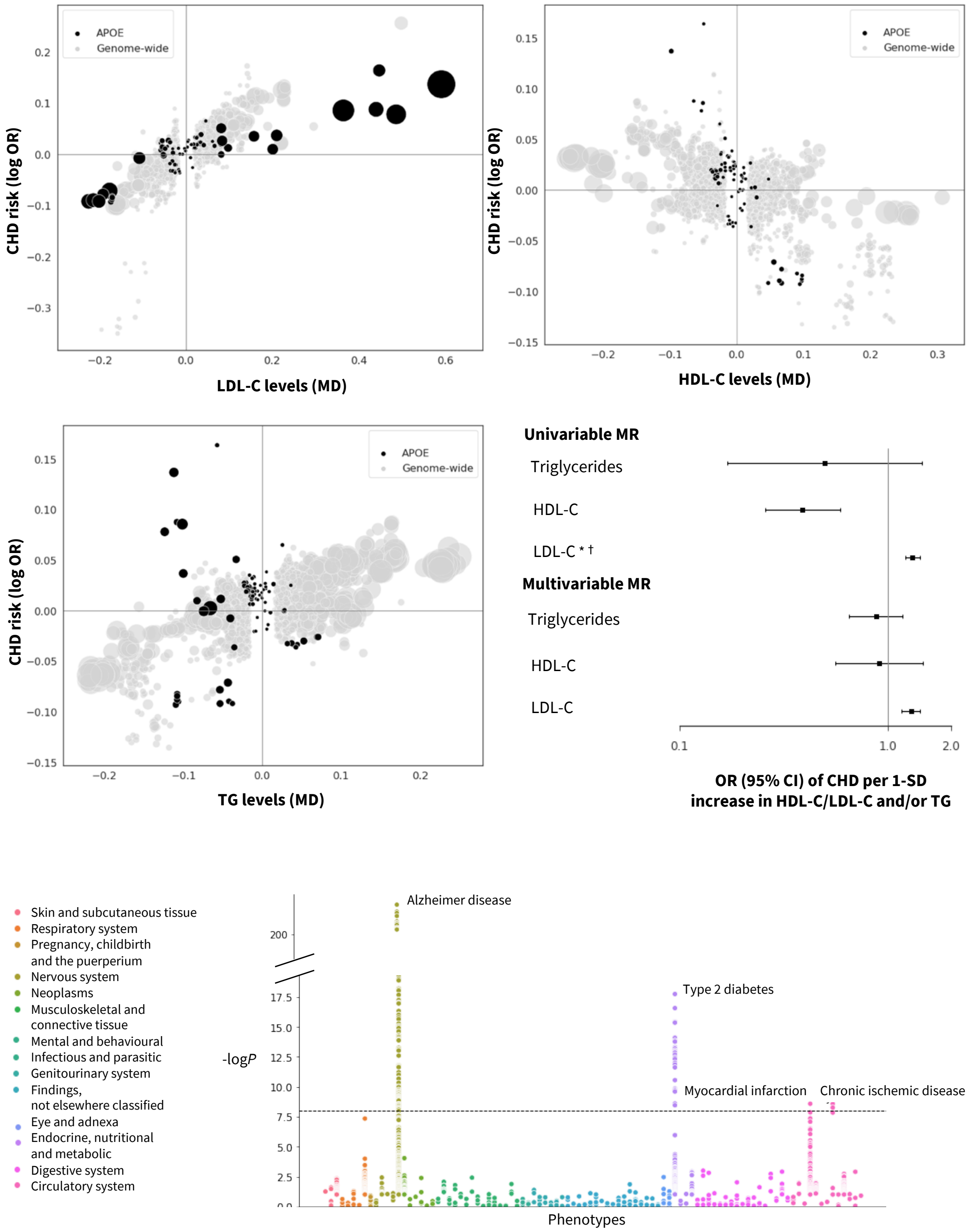

Supplementary Figure 20. Prioritized target: APOE. The top and middle left panels show genetic associations at the locus (± 50kbp) in black vs genome-wide associations (grey, *P* value < $1x10^{-6}$ based on two-sided z-tests). The x-axis shows the per allele effect on the corresponding lipid expressed as mean difference (MD) from GLGC and the y-axis indicates the per allele effect on CHD expressed as log odds ratios (OR) from CardiogramPlusC4D. The marker size indicates the significance of the association with the lipid sub-fraction (*P*-value). The middle right panel shows the result of the univariable and multivariable (drug target) *cis*-MR results presented as OR and 95% confidence intervals with lipid exposure (n=188,577 individuals) and CHD outcome (n= 60,801 cases and 123,504 controls). '*' indicates the MR estimates as being replicated, and '†' that the lipid effect and CHD signals are co-localized. The bottom panel shows disease associations at the locus with 102 clinical end points from UK Biobank and GWAS Consortia.

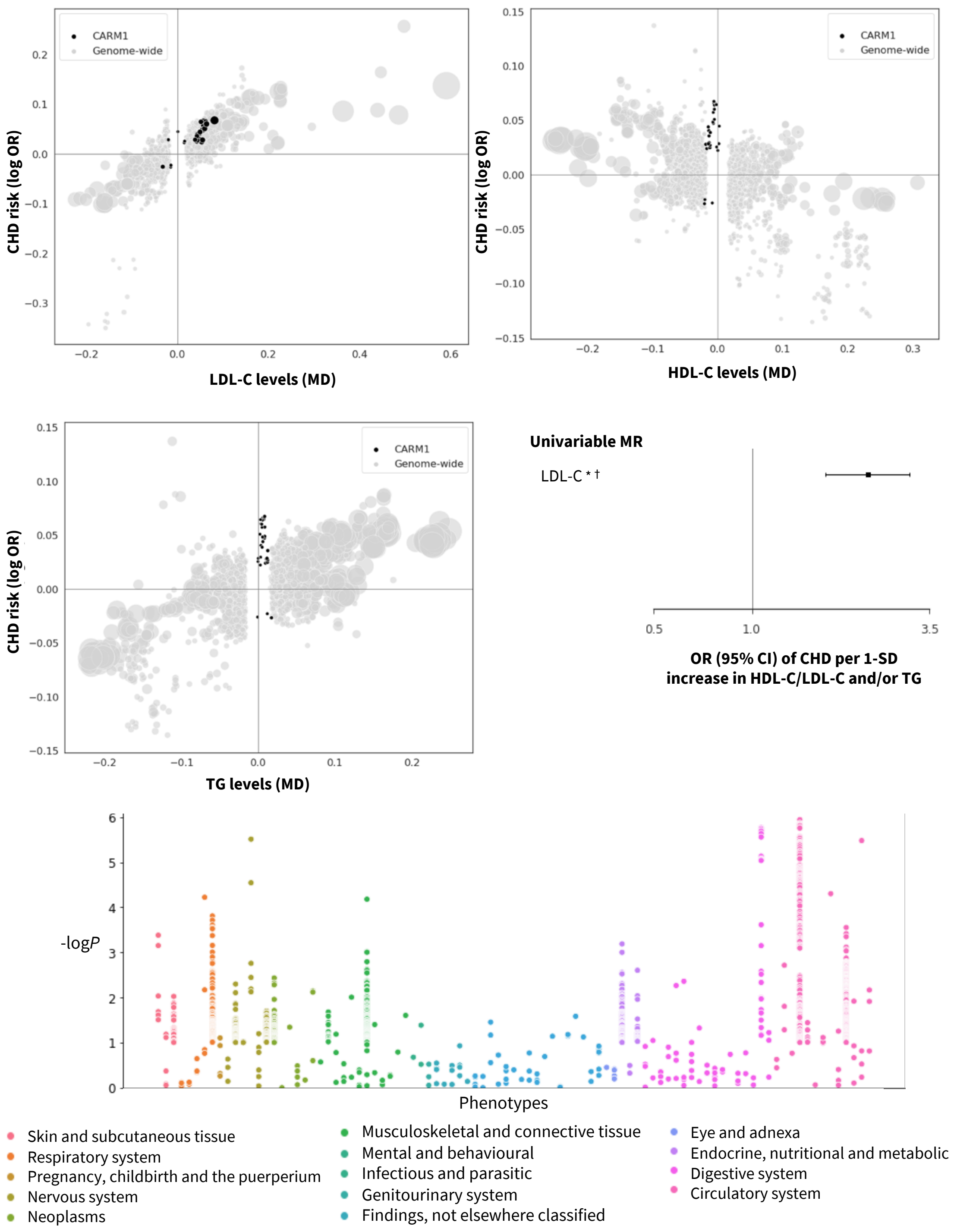

Supplementary Figure 21. Prioritized target: CARM1. The top and middle left panels show genetic associations at the locus (± 50kbp) in black vs genome-wide associations (grey, *P* value < $1x10^{-6}$ based on two-sided z-tests). The x-axis shows the per allele effect on the corresponding lipid expressed as mean difference (MD) from GLGC and the y-axis indicates the per allele effect on CHD expressed as log odds ratios (OR) from CardiogramPlusC4D. The marker size indicates the significance of the association with the lipid sub-fraction (*P*-value). The middle right panel shows the result of the univariable and multivariable (drug target) *cis*-MR results presented as OR and 95% confidence intervals with lipid exposure (n=188,577 individuals) and CHD outcome (n= 60,801 cases and 123,504 controls). '*' indicates the MR estimates as being replicated, and '†' that the lipid effect and CHD signals are co-localized. The bottom panel shows disease associations at the locus with 102 clinical end points from UK Biobank and GWAS Consortia.

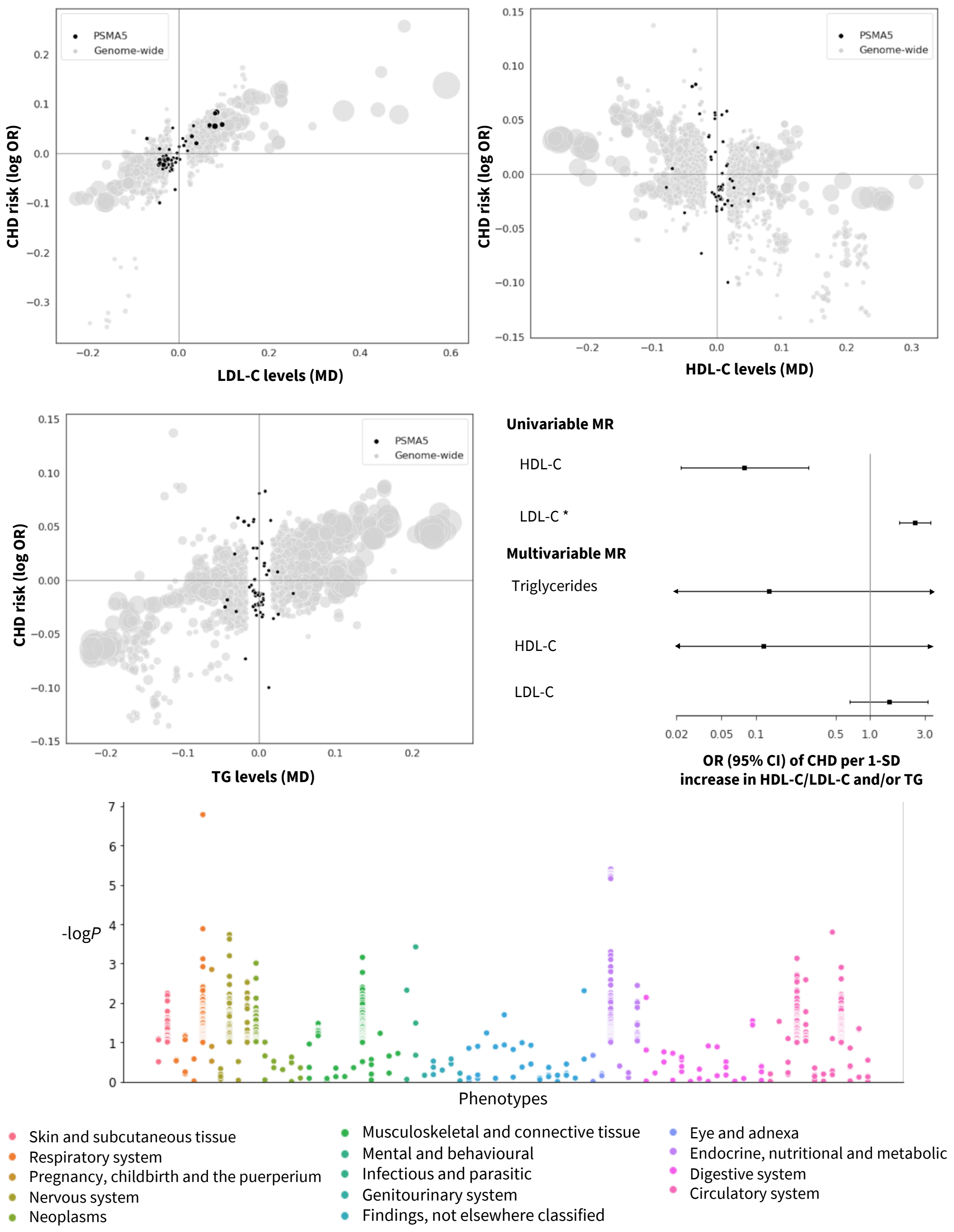

Supplementary Figure 22. Prioritized target: PSMA5. The top and middle left panels show genetic associations at the locus (± 50kbp) in black vs genome-wide associations (grey, *P* value < 1×10^{-6} based on two-sided z-tests). The x-axis shows the per allele effect on the corresponding lipid expressed as mean difference (MD) from GLGC and the y-axis indicates the per allele effect on CHD expressed as log odds ratios (OR) from CardiogramPlusC4D. The marker size indicates the significance of the association with the lipid sub-fraction (*P*-value). The middle right panel shows the result of the univariable and multivariable (drug target) *cis*-MR results presented as OR and 95% confidence intervals with lipid exposure (n=188,577 individuals) and CHD outcome (n= 60,801 cases and 123,504 controls). '*' indicates the MR estimates as being replicated, and '†' that the lipid effect and CHD signals are co-localized. The bottom panel shows disease associations at the locus with 102 clinical end points from UK Biobank and GWAS Consortia.

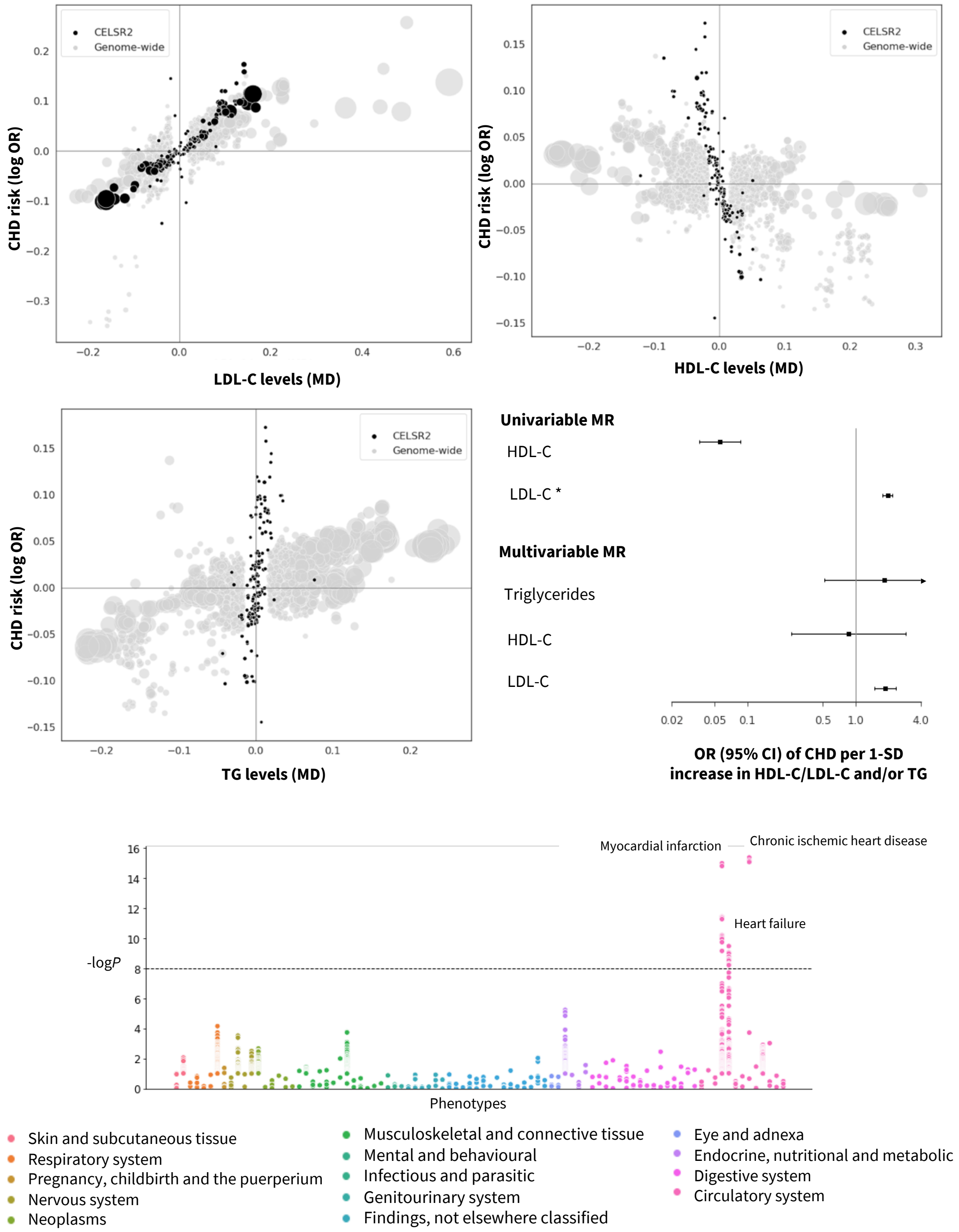

Supplementary Figure 23. Prioritized target: CELSR2. The top and middle left panels show genetic associations at the locus (± 50kbp) in black vs genome-wide associations (grey, *P* value < $1x10^{-6}$ based on two-sided z-tests). The x-axis shows the per allele effect on the corresponding lipid expressed as mean difference (MD) from GLGC and the y-axis indicates the per allele effect on CHD expressed as log odds ratios (OR) from CardiogramPlusC4D. The marker size indicates the significance of the association with the lipid sub-fraction (*P*-value). The middle right panel shows the result of the univariable and multivariable (drug target) *cis*-MR results presented as OR and 95% confidence intervals with lipid exposure (n=188,577 individuals) and CHD outcome (n= 60,801 cases and 123,504 controls). '*' indicates the MR estimates as being replicated, and '†' that the lipid effect and CHD signals are co-localized. The bottom panel shows disease associations at the locus with 102 clinical end points from UK Biobank and GWAS Consortia.

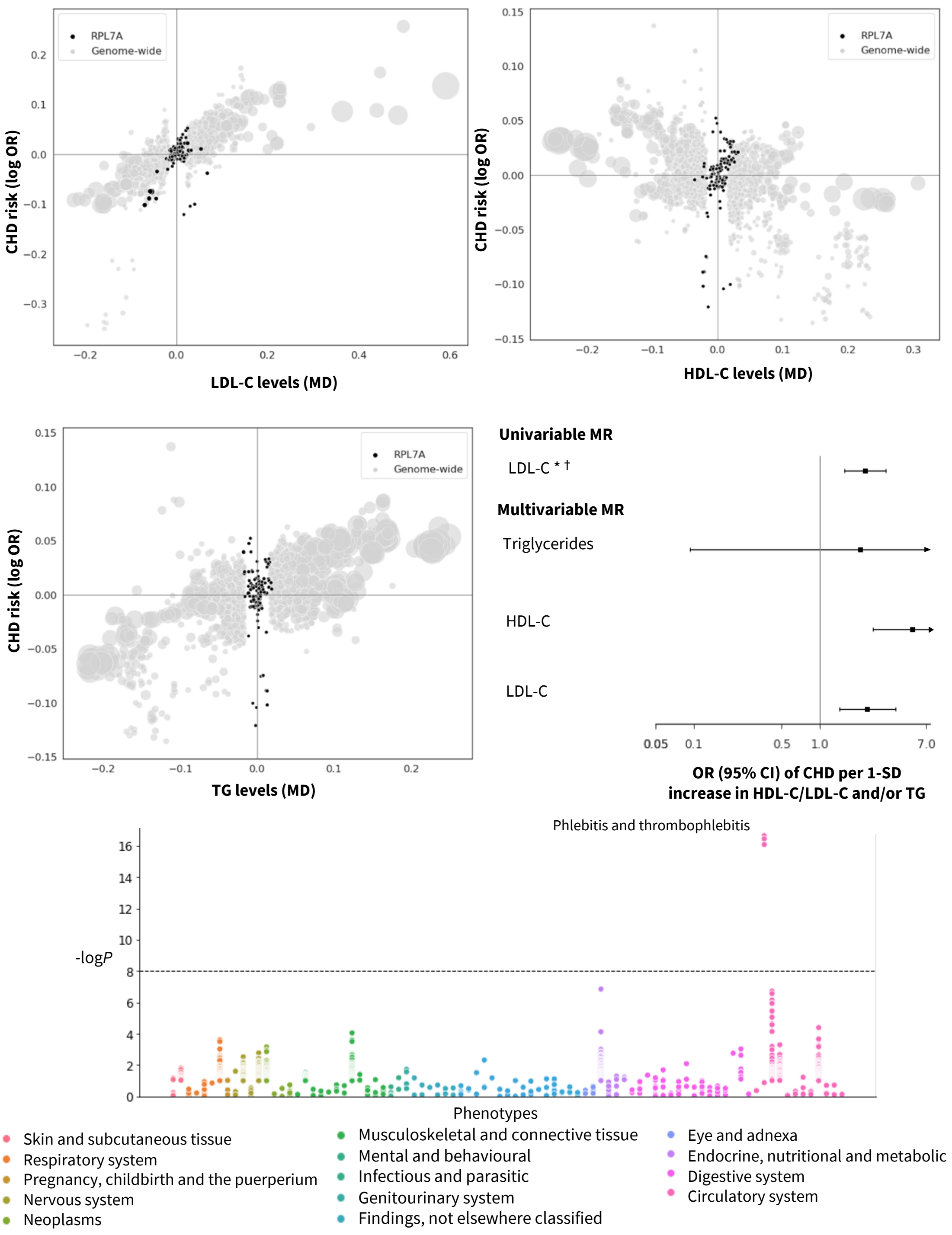

Supplementary Figure 24. Prioritized target: RPL7A. The top and middle left panels show genetic associations at the locus (± 50kbp) in black vs genome-wide associations (grey, *P* value < 1x10^{-6} based on two-sided z-tests). The x-axis shows the per allele effect on the corresponding lipid expressed as mean difference (MD) from GLGC and the y-axis indicates the per allele effect on CHD expressed as log odds ratios (OR) from CardiogramPlusC4D. The marker size indicates the significance of the association with the lipid sub-fraction (*P*-value). The middle right panel shows the result of the univariable and multivariable (drug target) *cis*-MR results presented as OR and 95% confidence intervals with lipid exposure (n=188,577 individuals) and CHD outcome (n= 60,801 cases and 123,504 controls). '*' indicates the MR estimates as being replicated, and '†' that the lipid effect and CHD signals are co-localized. The bottom panel shows disease associations at the locus with 102 clinical end points from UK Biobank and GWAS Consortia.

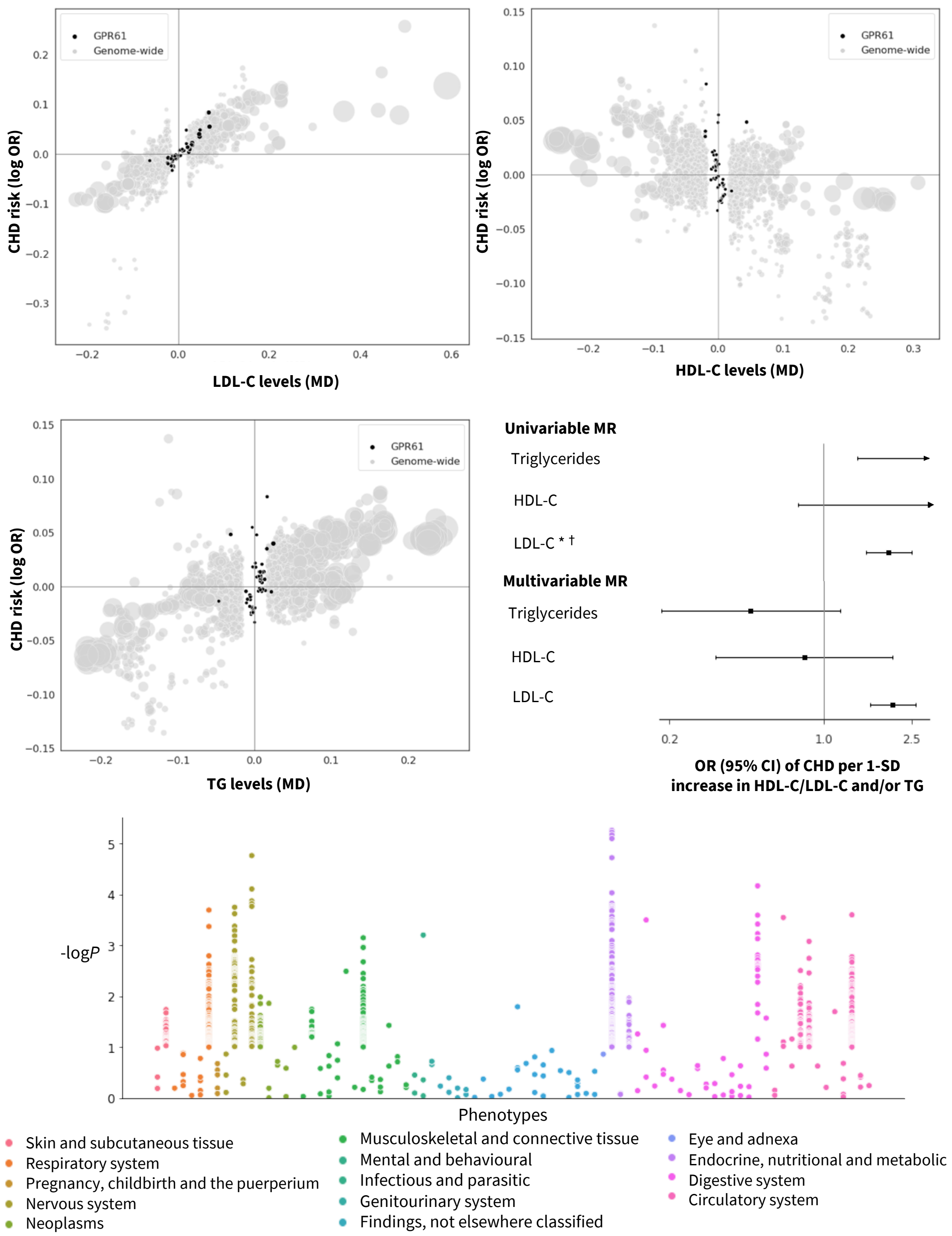

Supplementary Figure 25. Prioritized target: GPR61. The top and middle left panels show genetic associations at the locus (± 50kbp) in black vs genome-wide associations (grey, *P* value < 1×10^{-6} based on two-sided z-tests). The x-axis shows the per allele effect on the corresponding lipid expressed as mean difference (MD) from GLGC and the y-axis indicates the per allele effect on CHD expressed as log odds ratios (OR) from CardiogramPlusC4D. The marker size indicates the significance of the association with the lipid sub-fraction (*P*-value). The middle right panel shows the result of the univariable and multivariable (drug target) *cis*-MR results presented as OR and 95% confidence intervals with lipid exposure (n=188,577 individuals) and CHD outcome (n= 60,801 cases and 123,504 controls). '*' indicates the MR estimates as being replicated, and '†' that the lipid effect and CHD signals are co-localized. The bottom panel shows disease associations at the locus with 102 clinical end points from UK Biobank and GWAS Consortia..

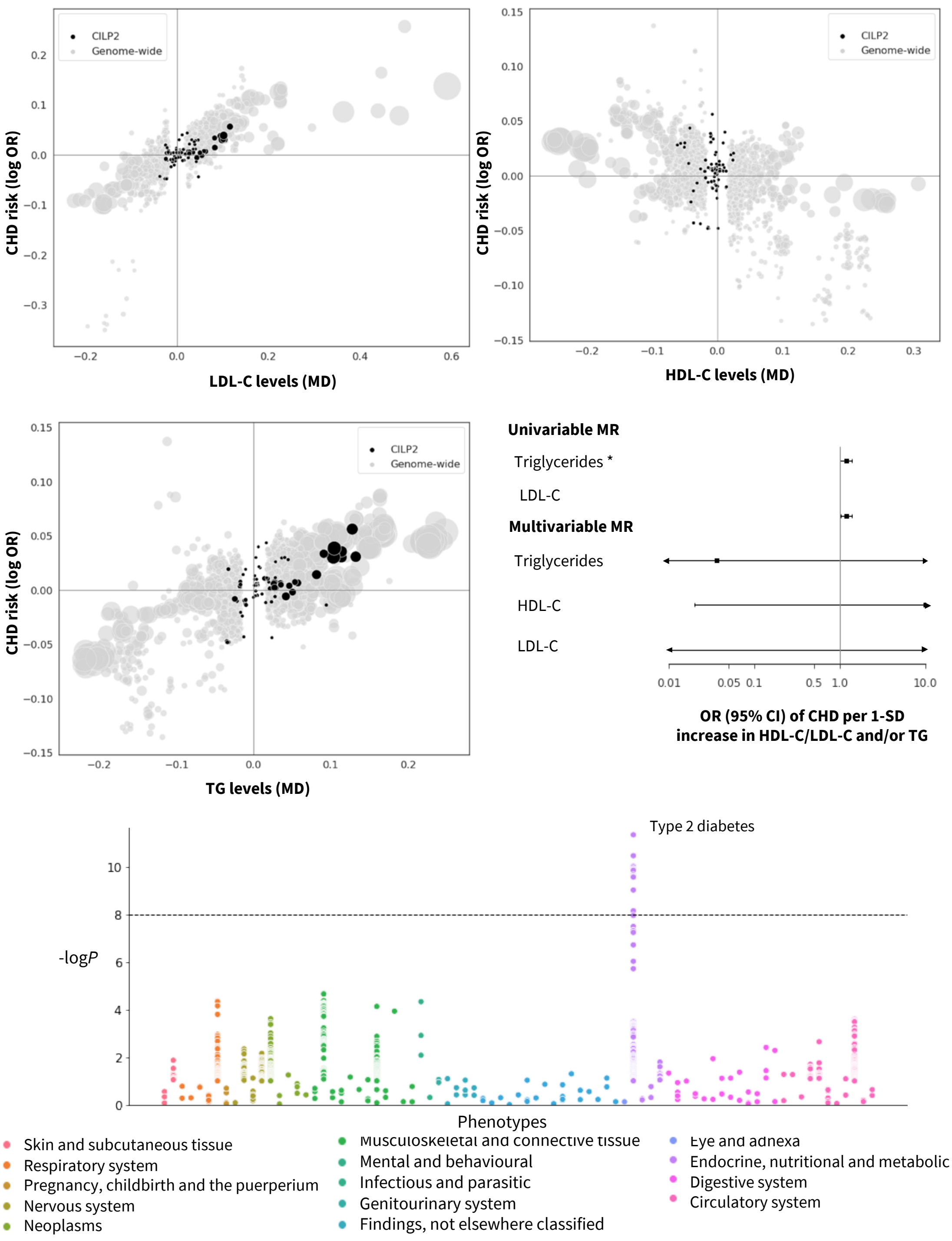

Supplementary Figure 26. Prioritized target: CILP2. The top and middle left panels show genetic associations at the locus (± 50kbp) in black vs genome-wide associations (grey, *P* value < $1x10^{-6}$ based on two-sided z-tests). The x-axis shows the per allele effect on the corresponding lipid expressed as mean difference (MD) from GLGC and the y-axis indicates the per allele effect on CHD expressed as log odds ratios (OR) from CardiogramPlusC4D. The marker size indicates the significance of the association with the lipid sub-fraction (*P*-value). The middle right panel shows the result of the univariable and multivariable (drug target) *cis*-MR results presented as OR and 95% confidence intervals with lipid exposure (n=188,577 individuals) and CHD outcome (n= 60,801 cases and 123,504 controls). '*' indicates the MR estimates as being replicated, and '†' that the lipid effect and CHD signals are co-localized. The bottom panel shows disease associations at the locus with 102 clinical end points from UK Biobank and GWAS Consortia.

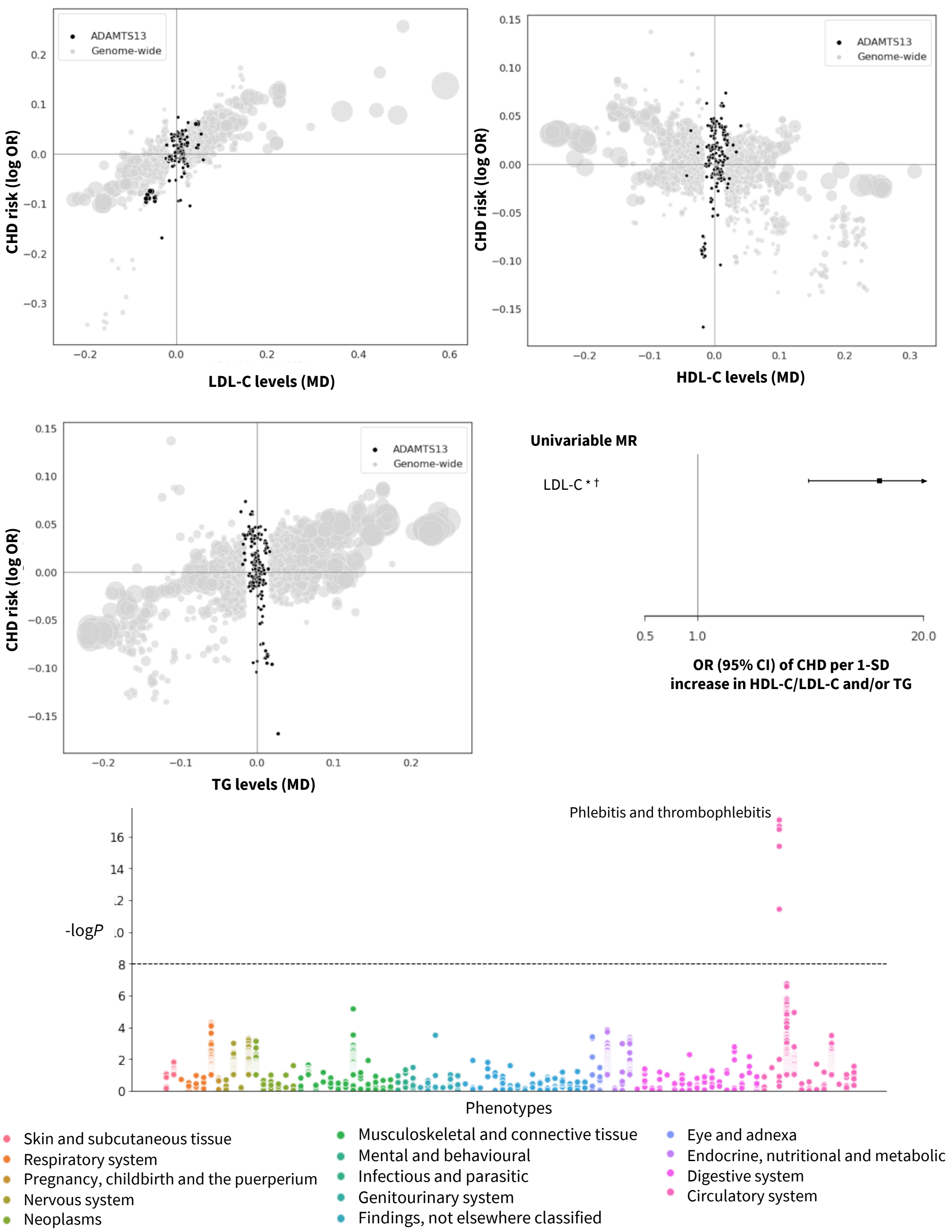

Supplementary Figure 27. Prioritized target: ADAMTS13. The top and middle left panels show genetic associations at the locus (± 50kbp) in black vs genome-wide associations (grey, *P* value < $1x10^{-6}$ based on two-sided z-tests). The x-axis shows the per allele effect on the corresponding lipid expressed as mean difference (MD) from GLGC and the y-axis indicates the per allele effect on CHD expressed as log odds ratios (OR) from CardiogramPlusC4D. The marker size indicates the significance of the association with the lipid sub-fraction (*P*-value). The middle right panel shows the result of the univariable and multivariable (drug target) *cis*-MR results presented as OR and 95% confidence intervals with lipid exposure (n=188,577 individuals) and CHD outcome (n= 60,801 cases and 123,504 controls). '*' indicates the MR estimates as being replicated, and '†' that the lipid effect and CHD signals are co-localized. The bottom panel shows disease associations at the locus with 102 clinical end points from UK Biobank and GWAS Consortia.

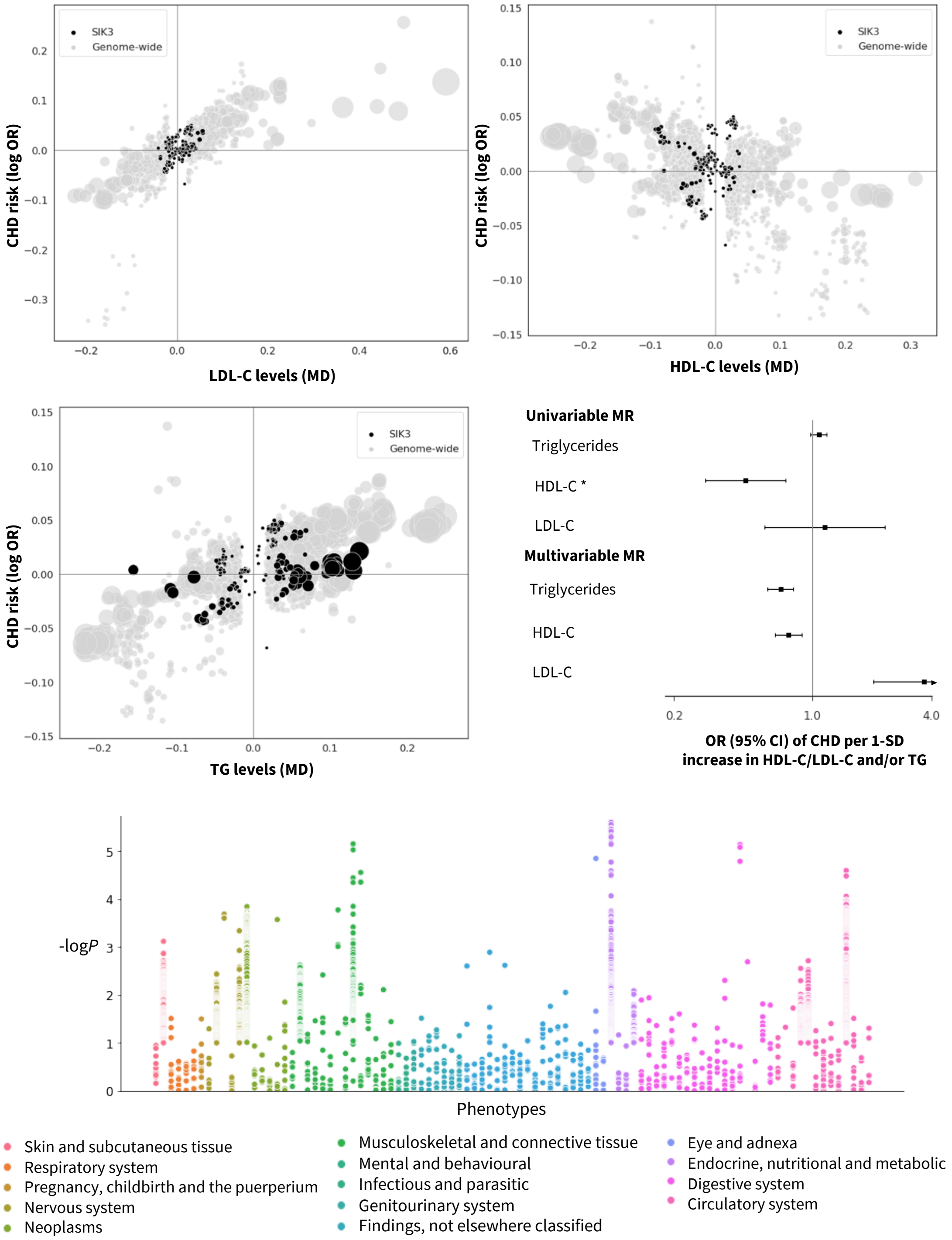

Supplementary Figure 28. Prioritized target: SIK3. The top and middle left panels show genetic associations at the locus (± 50kbp) in black vs genome-wide associations (grey, *P* value < $1x10^{-6}$ based on two-sided z-tests). The x-axis shows the per allele effect on the corresponding lipid expressed as mean difference (MD) from GLGC and the y-axis indicates the per allele effect on CHD expressed as log odds ratios (OR) from CardiogramPlusC4D. The marker size indicates the significance of the association with the lipid sub-fraction (*P*-value). The middle right panel shows the result of the univariable and multivariable (drug target) *cis*-MR results presented as OR and 95% confidence intervals with lipid exposure (n=188,577 individuals) and CHD outcome (n= 60,801 cases and 123,504 controls). '*' indicates the MR estimates as being replicated, and '†' that the lipid effect and CHD signals are co-localized. The bottom panel shows disease associations at the locus with 102 clinical end points from UK Biobank and GWAS Consortia.

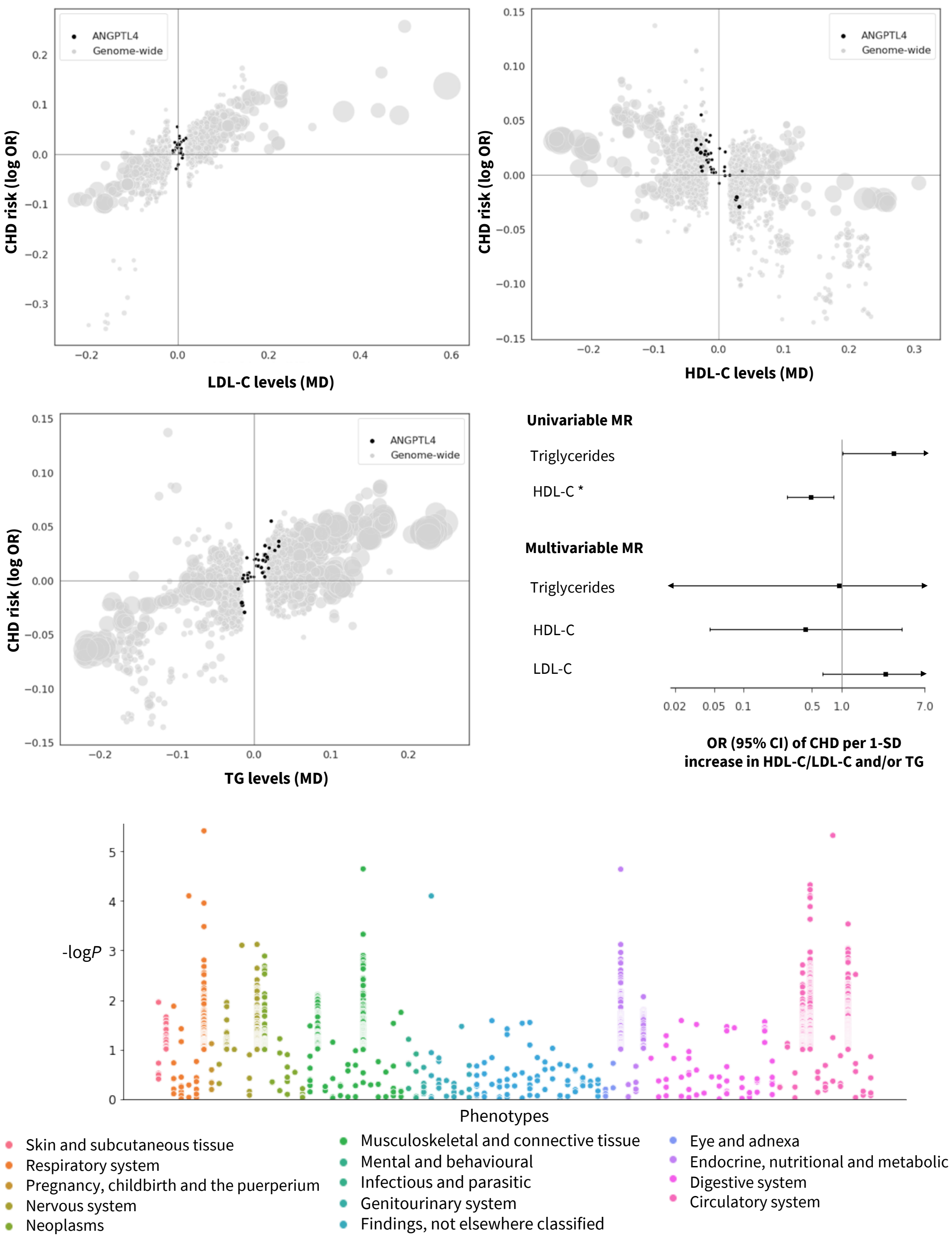

Supplementary Figure 29. Prioritized target: ANGPTL4. The top and middle left panels show genetic associations at the locus (± 50kbp) in black vs genome-wide associations (grey, *P* value < $1x10^{-6}$ based on two-sided z-tests). The x-axis shows the per allele effect on the corresponding lipid expressed as mean difference (MD) from GLGC and the y-axis indicates the per allele effect on CHD expressed as log odds ratios (OR) from CardiogramPlusC4D. The marker size indicates the significance of the association with the lipid sub-fraction (*P*-value). The middle right panel shows the result of the univariable and multivariable (drug target) *cis*-MR results presented as OR and 95% confidence intervals with lipid exposure (n=188,577 individuals) and CHD outcome (n= 60,801 cases and 123,504 controls). '*' indicates the MR estimates as being replicated, and '†' that the lipid effect and CHD signals are co-localized. The bottom panel shows disease associations at the locus with 102 clinical end points from UK Biobank and GWAS Consortia.

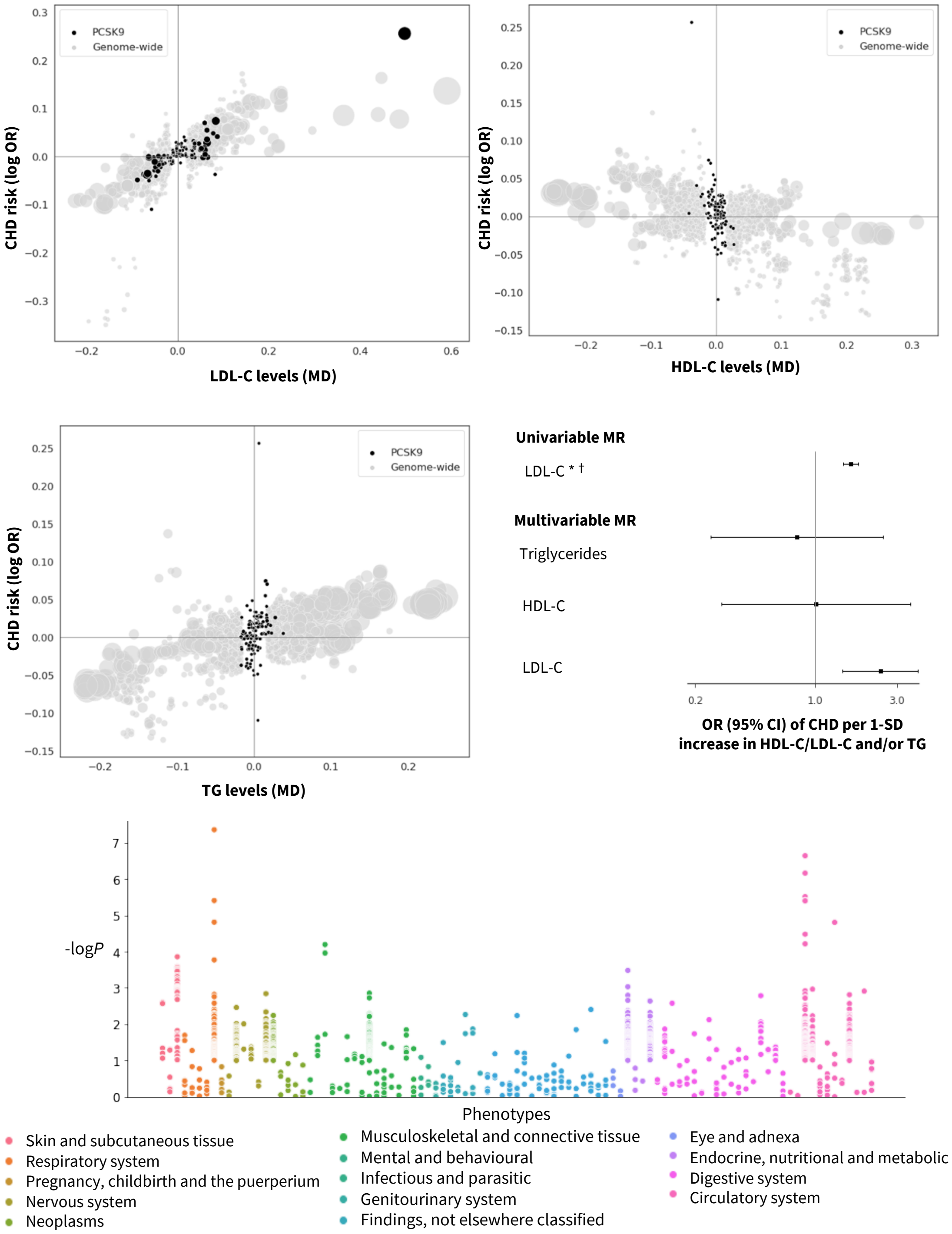

Supplementary Figure 30. Prioritized target: PCSK9. The top and middle left panels show genetic associations at the locus (± 50kbp) in black vs genome-wide associations (grey, *P* value < $1x10^{-6}$ based on two-sided z-tests). The x-axis shows the per allele effect on the corresponding lipid expressed as mean difference (MD) from GLGC and the y-axis indicates the per allele effect on CHD expressed as log odds ratios (OR) from CardiogramPlusC4D. The marker size indicates the significance of the association with the lipid sub-fraction (*P*-value). The middle right panel shows the result of the univariable and multivariable (drug target) *cis*-MR results presented as OR and 95% confidence intervals with lipid exposure (n=188,577 individuals) and CHD outcome (n= 60,801 cases and 123,504 controls). '*' indicates the MR estimates as being replicated, and '†' that the lipid effect and CHD signals are co-localized. The bottom panel shows disease associations at the locus with 102 clinical end points from UK Biobank and GWAS Consortia.

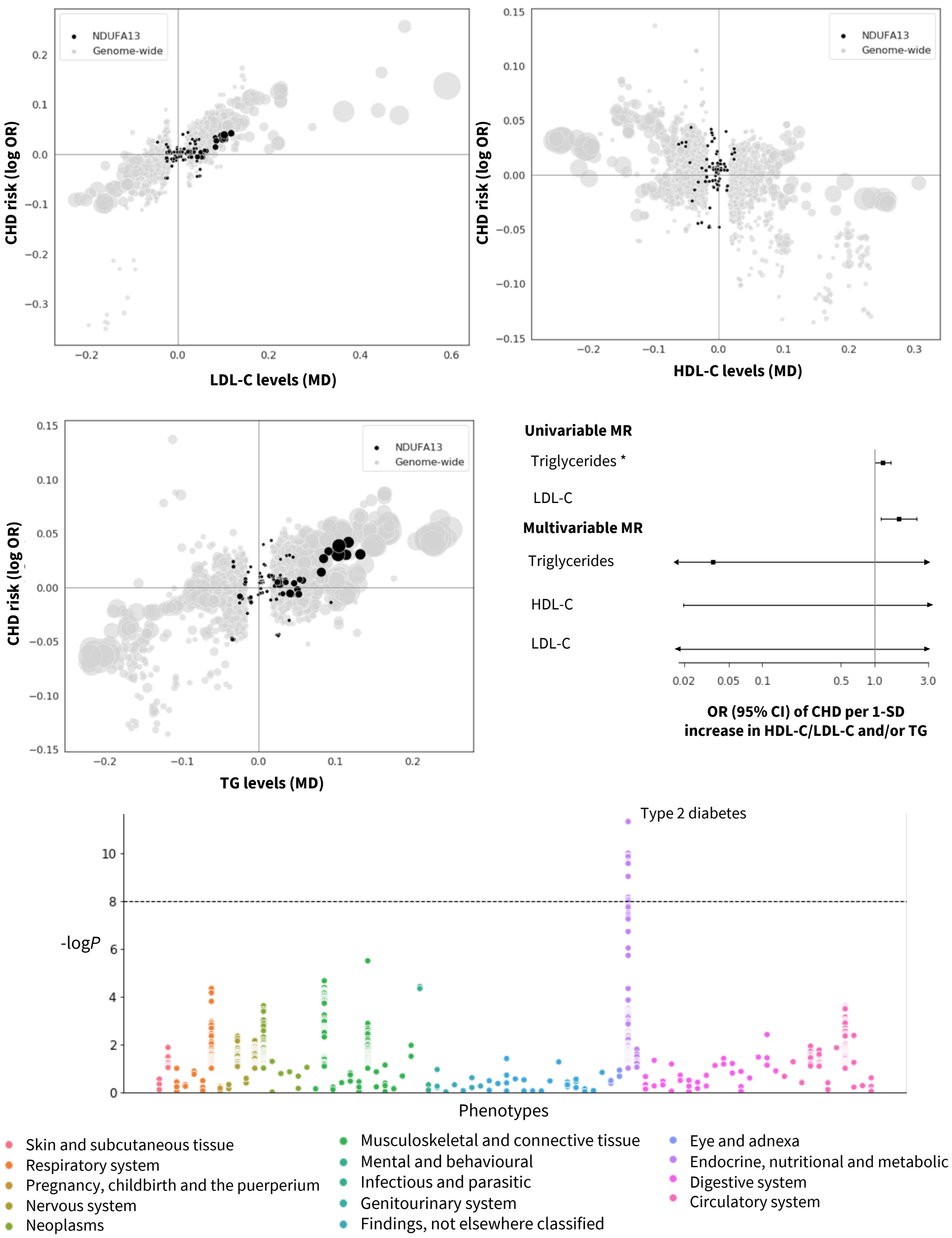

Supplementary Figure 31. Prioritized target: NDUFA13. The top and middle left panels show genetic associations at the locus (± 50kbp) in black vs genome-wide associations (grey, *P* value < $1x10^{-6}$ based on two-sided z-tests). The x-axis shows the per allele effect on the corresponding lipid expressed as mean difference (MD) from GLGC and the y-axis indicates the per allele effect on CHD expressed as log odds ratios (OR) from CardiogramPlusC4D. The marker size indicates the significance of the association with the lipid sub-fraction (*P*-value). The middle right panel shows the result of the univariable and multivariable (drug target) *cis*-MR results presented as OR and 95% confidence intervals with lipid exposure (n=188,577 individuals) and CHD outcome (n= 60,801 cases and 123,504 controls). '*' indicates the MR estimates as being replicated, and '†' that the lipid effect and CHD signals are co-localized. The bottom panel shows disease associations at the locus with 102 clinical end points from UK Biobank and GWAS Consortia.

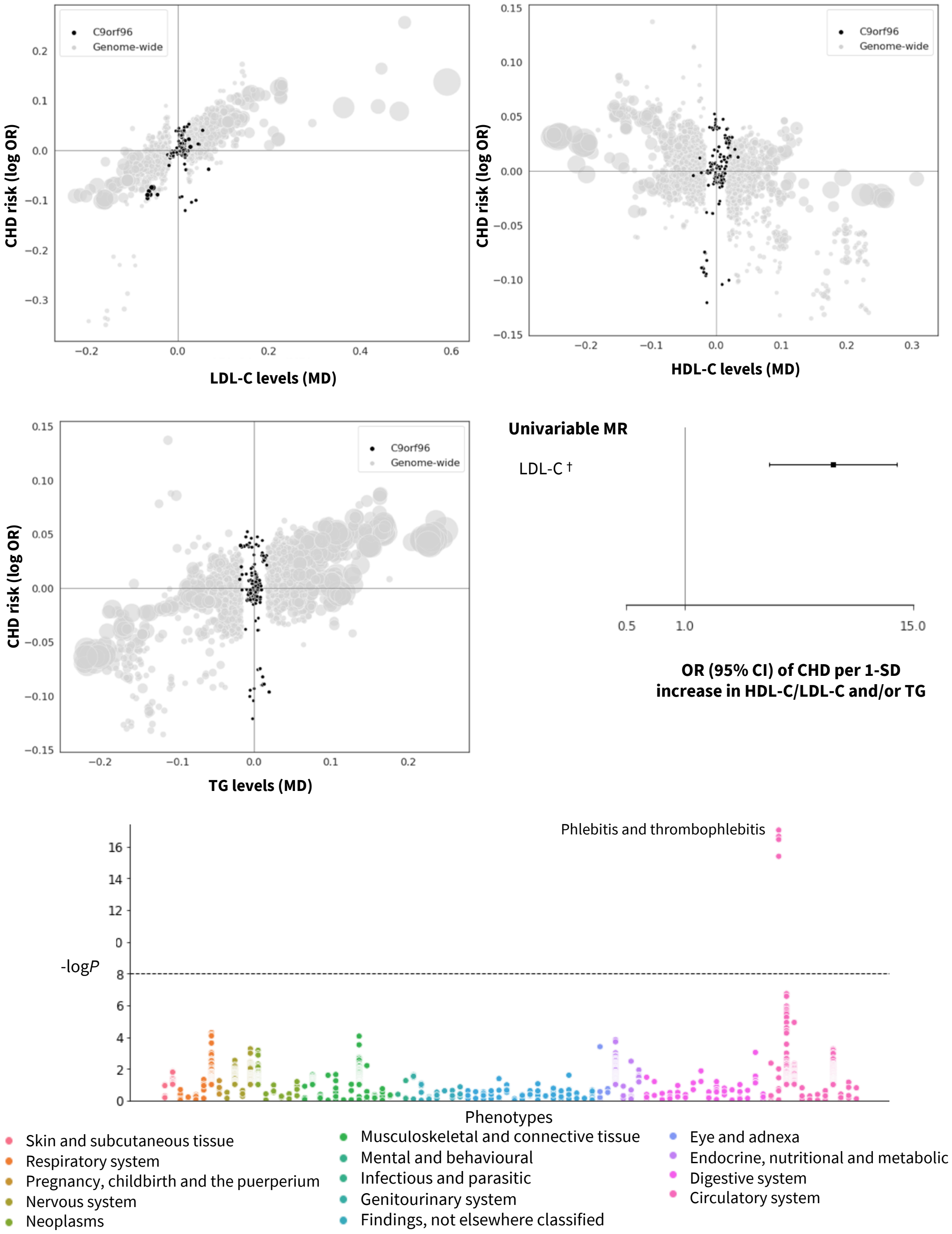

Supplementary Figure 32. Prioritized target: C9orf96. The top and middle left panels show genetic associations at the locus (± 50kbp) in black vs genome-wide associations (grey, *P* value < $1x10^{-6}$ based on two-sided z-tests). The x-axis shows the per allele effect on the corresponding lipid expressed as mean difference (MD) from GLGC and the y-axis indicates the per allele effect on CHD expressed as log odds ratios (OR) from CardiogramPlusC4D. The marker size indicates the significance of the association with the lipid sub-fraction (*P*-value). The middle right panel shows the result of the univariable and multivariable (drug target) *cis*-MR results presented as OR and 95% confidence intervals with lipid exposure (n=188,577 individuals) and CHD outcome (n= 60,801 cases and 123,504 controls). '*' indicates the MR estimates as being replicated, and '†' that the lipid effect and CHD signals are co-localized. The bottom panel shows disease associations at the locus with 102 clinical end points from UK Biobank and GWAS Consortia.

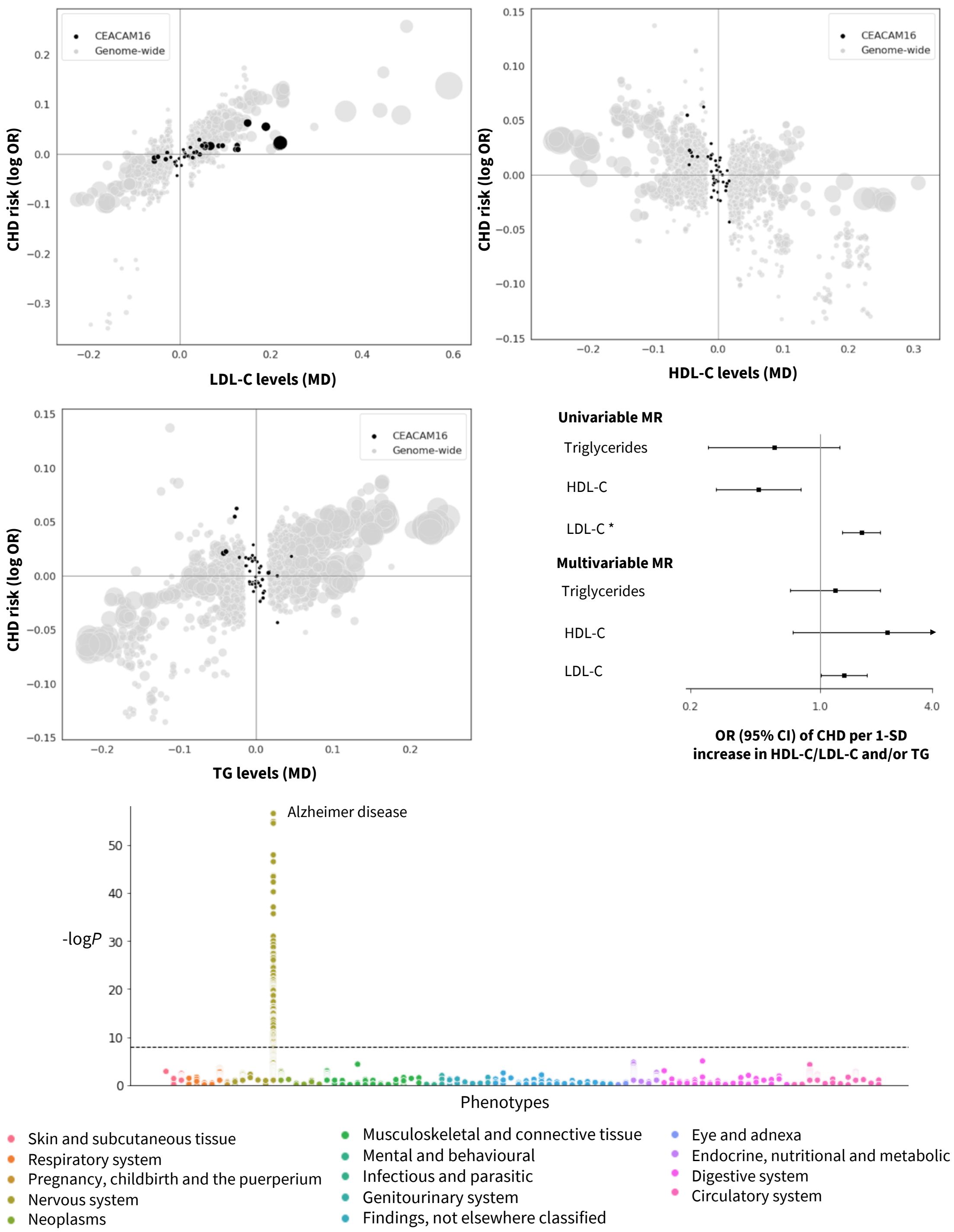

Supplementary Figure 33. Prioritized target: CEACAM16. The top and middle left panels show genetic associations at the locus (± 50kbp) in black vs genome-wide associations (grey, P value < $1x10^{-6}$ based on two-sided z-tests). The x-axis shows the per allele effect on the corresponding lipid expressed as mean difference (MD) from GLGC and the y-axis indicates the per allele effect on CHD expressed as log odds ratios (OR) from CardiogramPlusC4D. The marker size indicates the significance of the association with the lipid sub-fraction (*P*-value). The middle right panel shows the result of the univariable and multivariable (drug target) *cis*-MR results presented as OR and 95% confidence intervals with lipid exposure (n=188,577 individuals) and CHD outcome (n= 60,801 cases and 123,504 controls). '*' indicates the MR estimates as being replicated, and '†' that the lipid effect and CHD signals are co-localized. The bottom panel shows disease associations at the locus with 102 clinical end points from UK Biobank and GWAS Consortia.

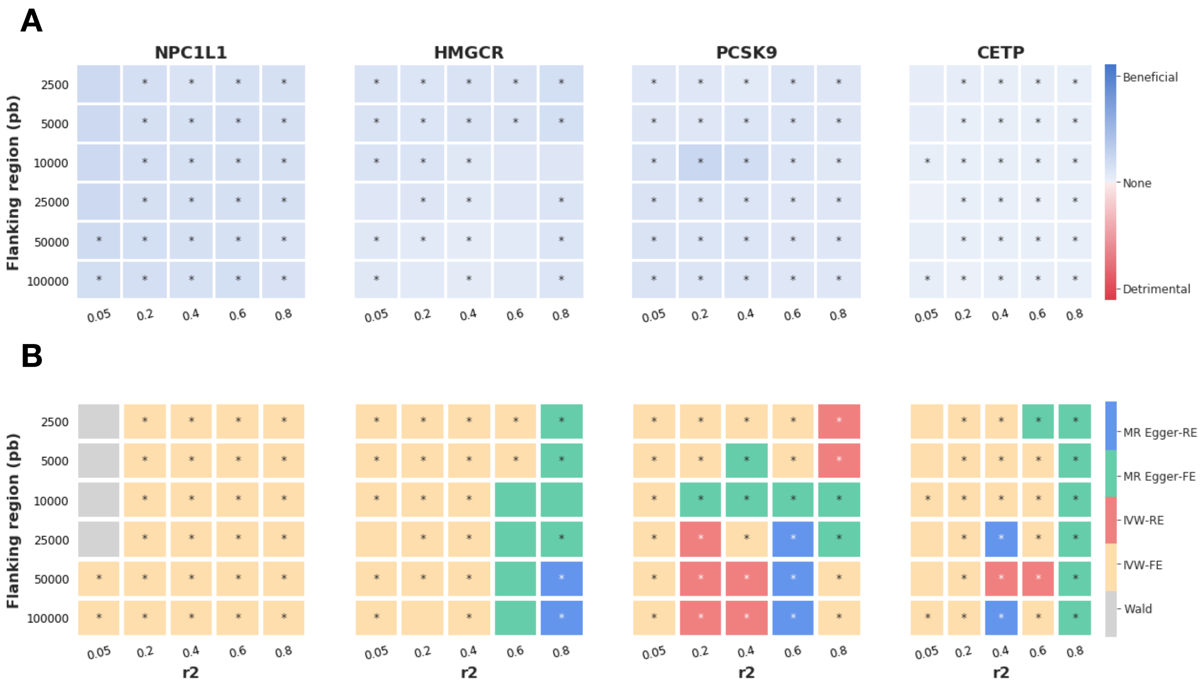

Supplementary Figure 34. Drug target MR of positive control examples. Grid search of LD threshold and region around the gene encoding a druggable target using genetic associations with LDL-C and HDL-C from the Global Lipid Genetic Consortium (GLGC) with CHD events from the CardiogramPlusC4D Consortium**.** MR estimates (**A**) and preferred model (**B**) for three licensed LDL-lowering drug targets and HDL-lowering CETP using lipid data from GLGC and CHD data from CardiogramPlusC4D in the discovery analysis. Models explored: MR Egger-RE (random effects), MR Egger-RE (fixed effects), inverse variance weighted (IVW)-RE (random effects), IVW-FE (fixed effects), Wald ratio.

Supplementary Table 1. Main conceptual differences between *genome-wide biomarker* and *drug target* MR approaches.

	Genome-wide biomarker MR	Drug target MR
**Aim**	Causal effect of a biomarker	Causal relevance of a drug target
**SNP selection**	Genome-wide	Locus specific
**Ideal exposure**	Clinically relevant quantitative trait	mRNA or protein expression of the encoded gene
**MR methods**	Any described MR method	Methods accounting for residual genetic correlation to maximize power

Supplementary Table 2. Causal odds ratios (95% CI) for CHD per standard deviation increase in each lipid sub-fraction from a biomarker MR analysis.

Method	LDL-C (OR, 95%CI)	HDL-C (OR, 95%CI)	Triglycerides (OR, 95%CI)	Ref.
Regression-based method	1.46 (1.37, 1.57) No. variants = 185	0.96 (0.89, 1.03) No. variants = 185	1.43 (1.28, 1.61) No. variants = 185	*(1)*
Multivariable IVW MR	1.48 (1.36, 1.61) No. variants = 185	0.93 (0.85, 1.02) No. variants = 185	1.16 (1.04, 1.29) No. variants = 185	(2)
Univariable MR-Egger regression	1.51 (1.30, 1.63) No. variants = 418	0.95 (0.90, 1.01) No. variants = 456	1.11 (1.01, 1.21) No. variants = 381	-
Multivariable MR-Egger regression	1.53 (1.44, 1.62) No. variants = 677	0.91 (0.86, 0.95) n = 677	1.09 (1.01, 1.17) No. variants = 677	-

All the studies used variants from the Global Lipid Genetic Consortium (GLGC) to instrument causal effects of LDL-C, HDL-C and triglycerides on CHD from the CardiogramPlusC4D Consortium. For details see text. OR = odds ratio per 1-SD increase in LDL-C/HDL-C or triglycerides; CI = confidence interval.

Supplementary Table 3. Publicly available GWAS data used in the phenome-wide association analysis (PheWAS).

Trait	No. Events	Sample size	URL
**Rheumatoid arthritis**	14361	57284	https://grasp.nhlbi.nih.gov/downloads/ResultsOctober2016/Okada/
**Juvenile arthritis**	2816	15872	http://ftp.ebi.ac.uk/pub/databases/gwas/summary_statistics/HinksA_23603761_GCST005528/
**Ankylosing spondylitis**	10619	15145	https://ftp.ebi.ac.uk/pub/databases/gwas/summary_statistics/CortesA_23749187_GCST005529/
**Ulcerative colitis**	6968	27432	http://ftp.ebi.ac.uk/pub/databases/gwas/summary_statistics/LiuJZ_26192919_GCST003045/
**Psoriasis**	10588	33394	http://ftp.ebi.ac.uk/pub/databases/gwas/summary_statistics/TsoiLC_23143594_GCST005527/
**Crohn disease**	22575	69268	http://ftp.ebi.ac.uk/pub/databases/gwas/summary_statistics/LiuJZ_26192919
**Stroke**	34217	440328	http://www.megastroke.org/index.html
**Asthma**	5135	30810	https://www.thelancet.com/journals/lanres/article/PIIS2213-2600(18)30389-8/fulltext
**Multiple sclerosis**	14498	38589	https://www.ncbi.nlm.nih.gov/pmc/articles/PMC3832895/
**Gout**	13179	882413	http://ftp.ebi.ac.uk/pub/databases/gwas/summary_statistics/TinA_31578528_GCST008970/
**Ovarian neoplasms**	16924	85426	http://ftp.ebi.ac.uk/pub/databases/gwas/summary_statistics/PhelanCM_28346442_GCST004462/
**Parkinson disease**	15056	27693	https://drive.google.com/drive/folders/10bGj6HfAXgl-JslpI9ZJIL_JIgZyktxn
**Alzheimer disease**	71880	455258	https://www.ncbi.nlm.nih.gov/pubmed/30617256
**Type 2 diabetes mellitus**	74124	898130	https://www.nature.com/articles/s41588-018-0241-6
**Myocardial infarction**	40149	126310	http://www.cardiogramplusc4d.org/data-downloads/
**Heart failure**	47309	977323	https://www.nature.com/articles/s41467-019-13690-5
**Atrial fibrillation**	60620	1030836	https://www.nature.com/articles/s41588-018-0171-3
**Diabetic nephropathies**	5908	10875	http://ftp.ebi.ac.uk/pub/databases/gwas/summary_statistics/vanZuydamNR_29703844_GCST005881
**Chronic kidney failure**	64164	625219	https://www.ncbi.nlm.nih.gov/pmc/articles/PMC6698888/
**Schizophrenia**	35476	46839	http://www.med.unc.edu/pgc/files/resultfiles/
**Narcolepsy**	1886	12307	http://ftp.ebi.ac.uk/pub/databases/gwas/summary_statistics/FaracoJ_23459209_GCST005522/
**Atopic dermatitis**	21399	95464	http://ftp.ebi.ac.uk/pub/databases/gwas/summary_statistics/PaternosterL_26482879_GCST003184
**Biliary liver cirrhosis**	2764	13239	http://ftp.ebi.ac.uk/pub/databases/gwas/summary_statistics/CordellHJ_26394269_GCST003129
