# Supplementary material for: Validation of lipid-related therapeutic targets for coronary heart disease prevention using human genetics
Source: Nat Commun. 2021 Oct 21;12:6120. doi: 10.1038/s41467-021-25731-z (PMC8531035; doi:10.1038/s41467-021-25731-z)
Supplement: Supplementary file 3 — Description of additional supplementary files [file 41467_2021_25731_MOESM3_ESM.docx]

**Description of additional supplementary files**

**File name: Supplementary Data 1**

**Description**: Proximity to GWAS SNP, distance rank and previous evidence of druggable genes near genetic associations with LDL-C, HDL-C and TG. For each druggable gene included in the analysis, the minimum distance from the gene to the variant (variants located within a gene were given a distance of 0bp and distance to variants upstream the gene are indicated with a negative value), a gene distance rank value according to their base pair distance, and indicated the druggable genes prioritized by GLGC are provided. OR = odds ratio per 1-SD increase in LDL-C/HDL-C or triglycerides; CI = confidence interval.

**File name: Supplementary Data 2**

**Description**: Univariable drug target MR estimates in the discovery analysis. * indicates significant in the discovery analysis; † indicates significant in both original and validation study and concordant direction of effect. OR = odds ratio per 1-SD increase in LDL-C/HDL-C or triglycerides; CI = confidence interval.

**File name: Supplementary Data 3**

**Description**: Univariable MR estimates of drug targets with lipid records in clinicaltrials.gov and/or the British National Formulary (BNF). * indicates significance in the discovery analysis; † indicates significance in both original and validation study and concordant direction of effect. OR = CHD odds ratio per 1-SD increase in LDL-C/HDL-C or triglycerides; CI = confidence interval.

**File name: Supplementary Data 4**

**Description**: Multivariable drug target MR estimates. OR = CHD odds ratio per 1-SD increase in LDL-C/HDL-C or triglycerides; CI = confidence interval. An asterisk (*) indicates significant estimates.

**File name: Supplementary Data 5**

**Description:** Clinical endpoints interrogated in the phenome-wide association analysis (PheWAS).
