# Supplementary material for: Validation of lipid-related therapeutic targets for coronary heart disease prevention using human genetics
Source: Nat Commun. 2021 Oct 21;12:6120. doi: 10.1038/s41467-021-25731-z (PMC8531035; doi:10.1038/s41467-021-25731-z)
Supplement: Supplementary file 9 — Reporting Summary [file 41467_2021_25731_MOESM9_ESM.pdf]

nature research

Corresponding author(s): María Gordillo-Marañón

Last updated by author(s): 08/10/2021

Reporting Summary

Nature Research wishes to improve the reproducibility of the work that we publish. This form provides structure for consistency and transparency in reporting. For further information on Nature Research policies, see our Editorial Policies and the Editorial Policy Checklist.

Statistics

For all statistical analyses, confirm that the following items are present in the figure legend, table legend, main text, or Methods section.

n/a	Confirmed	
☐	☒	The exact sample size (n) for each experimental group/condition, given as a discrete number and unit of measurement
☐	☒	A statement on whether measurements were taken from distinct samples or whether the same sample was measured repeatedly
☐	☒	The statistical test(s) used AND whether they are one- or two-sided<br>*Only common tests should be described solely by name; describe more complex techniques in the Methods section.*
☒	☐	A description of all covariates tested
☐	☒	A description of any assumptions or corrections, such as tests of normality and adjustment for multiple comparisons
☐	☒	A full description of the statistical parameters including central tendency (e.g. means) or other basic estimates (e.g. regression coefficient) AND variation (e.g. standard deviation) or associated estimates of uncertainty (e.g. confidence intervals)
☐	☒	For null hypothesis testing, the test statistic (e.g. F, t, r) with confidence intervals, effect sizes, degrees of freedom and P value noted<br>*Give P values as exact values whenever suitable.*
☐	☒	For Bayesian analysis, information on the choice of priors and Markov chain Monte Carlo settings
☒	☐	For hierarchical and complex designs, identification of the appropriate level for tests and full reporting of outcomes
☐	☒	Estimates of effect sizes (e.g. Cohen's d, Pearson's r), indicating how they were calculated

Our web collection on statistics for biologists contains articles on many of the points above.

Software and code

Policy information about availability of computer code

Data collection	Data were collected from publicly available repositories; no specialist software was used for this.
Data analysis	Mendelian Randomization analyses were conducted using Python v3.7.3. Co-localisation analyses were done using a stepwise conditional analysis using GCTA-COJO v1.92.4 and "coloc" v3.2-1.

For manuscripts utilizing custom algorithms or software that are central to the research but not yet described in published literature, software must be made available to editors and reviewers. We strongly encourage code deposition in a community repository (e.g. GitHub). See the Nature Research guidelines for submitting code & software for further information.

Data

Policy information about availability of data

All manuscripts must include a data availability statement. This statement should provide the following information, where applicable:

- Accession codes, unique identifiers, or web links for publicly available datasets
- A list of figures that have associated raw data
- A description of any restrictions on data availability

All source GWAS data used throughout the manuscript publicly available as specified in the Methods.

We used GWAS data for the following outcomes (Supplementary Table 7 and Supplementary Table 8): LDL-C, HDL-C, Triglycerides [http://lipidgenetics.org/#data-downloads-title], Coronary Heart Disease [http://www.cardiogramplusc4d.org/data-downloads/], Rheumatoid arthritis [https://grasp.nhlbi.nih.gov/downloads/ResultsOctober2016/Okada/], Juvenile arthritis [http://ftp.ebi.ac.uk/pub/databases/gwas/summary_statistics/HinksA_23603761_GCST005528/], Ankylosing spondylitis [https://ftp.ebi.ac.uk/pub/databases/gwas/summary_statistics/CortesA_23749187_GCST005529/], Ulcerative colitis [http://ftp.ebi.ac.uk/pub/databases/gwas/summary_statistics/LiuJZ_26192919_GCST003045/], Psoriasis [http://ftp.ebi.ac.uk/pub/databases/gwas/summary_statistics/

TsoiLC_23143594_GCST005527/], Crohn disease [http://ftp.ebi.ac.uk/pub/databases/gwas/summary_statistics/LiuJZ_26192919], Stroke [http://www.megastroke.org/index.html], Asthma [https://www.thelancet.com/journals/lanres/article/PIIS2213-2600(18)30389-8/fulltext], Multiple sclerosis (https://www.ncbi.nlm.nih.gov/pmc/articles/PMC3832895/], Gout [http://ftp.ebi.ac.uk/pub/databases/gwas/summary_statistics/TinA_31578528_GCST008970/], Ovarian neoplasms [http://ftp.ebi.ac.uk/pub/databases/gwas/summary_statistics/PhelanCM_28346442_GCST004462/], Parkinson disease [https://drive.google.com/drive/folders/10bGj6HfAXgl-JslpI9ZJIL_JIgZyktxn], Alzheimer disease (https://www.ncbi.nlm.nih.gov/pubmed/30617256], Type 2 diabetes mellitus [https://www.nature.com/articles/s41588-018-0241-6], Myocardial infarction [http://www.cardiogramplusc4d.org/data-downloads/], Heart failure [https://www.nature.com/articles/s41467-019-13690-5], Atrial fibrillation [https://www.nature.com/articles/s41588-018-0171-3], Diabetic nephropathies [http://ftp.ebi.ac.uk/pub/databases/gwas/summary_statistics/vanZuydamNR_29703844_GCST005881], Chronic kidney failure [https://www.ncbi.nlm.nih.gov/pmc/articles/PMC6698888/], Schizophrenia [http://www.med.unc.edu/pgc/files/resultfiles/], Narcolepsy [http://ftp.ebi.ac.uk/pub/databases/gwas/summary_statistics/FaracoJ_23459209_GCST005522/], Atopic dermatitis [http://ftp.ebi.ac.uk/pub/databases/gwas/summary_statistics/PaternosterL_26482879_GCST003184], Biliary liver cirrhosis [http://ftp.ebi.ac.uk/pub/databases/gwas/summary_statistics/CordellHJ_26394269_GCST003129], and 80 ICD10 main diagnoses in UK Biobank released by Neale Lab (1st August 2018, http://www.nealelab.is/uk-biobank/). Data from clinical trials were queried from clinicaltrials.gov registry. Data on licensed drugs and compounds under development were sourced from the British National Formulary and ChEMBL v25 respectively.

The data underlying each figure have been deposited in the UCL Research Data Repository under accession code 10.5522/04/14555715 [http://dx.doi.org/10.5522/04/14555715.]

Field-specific reporting

Please select the one below that is the best fit for your research. If you are not sure, read the appropriate sections before making your selection.

[x] Life sciences [ ] Behavioural & social sciences [ ] Ecological, evolutionary & environmental sciences

For a reference copy of the document with all sections, see nature.com/documents/nr-reporting-summary-flat.pdf

Life sciences study design

All studies must disclose on these points even when the disclosure is negative.

Sample size	The manuscript uses publicly available data on genome-wide association studies. As such we did not perform any de novo participant recruitment, nor related sample size calculations. Precision of our results is indicated by 95% confidence intervals, where wide confidence intervals provide a clear indication that results can be improved by increasing sample size beyond the currently available number.
Data exclusions	To improve computational stability, variants with a low MAF (below 0.01) were excluded .This exclusion was pre-specified prior to the analysis.
Replication	Results were replicated in two independent datasets. The validation study replicated 83% of the initial estimates.
Randomization	Drug target Mendelian Randomization, uses genetic variants as instrumental variables, which, following Mendel law's of inheritance, are assumed to be randomly allocated during haplotype formation, resulting in a naturally occurring randomized experiment.
Blinding	Drug target Mendelian Randomization, uses genetic variants as instrumental variables, which, following Mendel law's of inheritance, are assumed to be randomly allocated during haplotype formation, resulting in a naturally occurring randomized experiment. In this setting the exposure of interest is the genetic variant in the gene encoding a drug target, and, in principle, neither the researcher or the subject were unaware of their genotype.

Reporting for specific materials, systems and methods

We require information from authors about some types of materials, experimental systems and methods used in many studies. Here, indicate whether each material, system or method listed is relevant to your study. If you are not sure if a list item applies to your research, read the appropriate section before selecting a response.

Materials & experimental systems

n/a	Involved in the study	
[x]	[ ]	Antibodies
[x]	[ ]	Eukaryotic cell lines
[x]	[ ]	Palaeontology and archaeology
[x]	[ ]	Animals and other organisms
[x]	[ ]	Human research participants
[x]	[ ]	Clinical data
[x]	[ ]	Dual use research of concern

Methods

n/a	Involved in the study	
[x]	[ ]	ChIP-seq
[x]	[ ]	Flow cytometry
[x]	[ ]	MRI-based neuroimaging
